# Adherence of Malaysian Adults’ Energy and Macronutrient Intakes to National Recommendations: A Review and Meta-Analysis

**DOI:** 10.3390/nu10111584

**Published:** 2018-10-28

**Authors:** Suzana Shahar, Hamid Jan Bin Jan Mohamed, Francisco de los Reyes, Maria Sofia Amarra

**Affiliations:** 1 Dietetic Programme and Centre of Healthy Aging and Wellness, Faculty of Health Sciences, Universiti Kebangsaan Malaysia, Jalan Raja Muda A. Aziz, 50300 Kuala Lumpur, Malaysia; suzana.shahar@ukm.edu.my; 2 Nutrition and Dietetics Program, School of Health Sciences, Universiti Sains Malaysia, 16150 Kubang Kerian, Kelantan, Malaysia; hamidjan@usm.my; 3 School of Statistics, University of the Philippines, Quirino Ave. cor. Kalaw St., Diliman, Quezon City 1101, Philippines; fndelosreyes@up.edu.ph; 4 International Life Sciences Institute Southeast Asia Region, 18 Mohamed Sultan Road, #03-01, Singapore 238967, Singapore; amarra.sofia@gmail.com

**Keywords:** adults, chronic disease, diet, macronutrients, energy, Malaysia

## Abstract

The present study examined the best available evidence regarding energy and macronutrient intake during adulthood (age 19 to 59 years) in Malaysia and assessed whether intakes adhere to national recommendations, in order to develop recommendations for dietary improvement based on population consumption patterns. A literature review and meta-analysis evaluated intake based on the following characteristics, using information from food balance sheets, national surveys, and individual studies: (1) levels of intake, (2) proportion of the population whose diets adhere to/exceed/fail to meet Malaysian Recommended Nutrient Intake (RNI) levels, and (3) sources of macronutrients observed in these studies. Food balance data suggested high levels of available energy, animal source protein, vegetable fat, and refined carbohydrates. Twenty studies (five nationwide, 15 individual) indicated that Malaysian adults generally met or exceeded recommendations for fat and protein, but were inconsistent with respect to energy and carbohydrates. Information on dietary sources was limited. Due to methodological limitations, insufficient evidence exists regarding energy and macronutrient intakes of Malaysian adults. Improved dietary assessment methods (including use of biomarkers), better data analysis, and updated food composition data, will provide more reliable information on which to base policy decisions and recommendations for improvement.

## 1. Introduction

Chronic disease development is age-related, and diet is an important risk factor for disease. Non-communicable diseases (NCDs) are fast becoming the major cause of morbidity and deaths in Malaysia [1], and dietary risk has been identified as the leading risk factor for disease and disability in the country [2]. The WHO Global Action Plan for Prevention and Control of NCDs 2013–2020 [3] identified voluntary targets for member countries, including a 25% reduction in risk of premature mortality from cardiovascular disease and other chronic diseases, by reducing modifiable risk factors such as unhealthy diets.

Malaysia uses its own Recommended Nutrient Intake (RNI) levels to evaluate dietary intakes of the population, identify risk of inadequate nutrient intakes for certain groups, and reduce risk of chronic diseases [4]. RNI is defined as the “daily intake, set at estimated average requirement (EAR) plus 2 standard deviations (SD), which meets the nutrient requirements of almost all (97.5%) apparently healthy individuals in an age- and sex-specific population group”. The 2017 RNI provides recommendations for energy (kcal/day) and protein (grams/day) intake based on reference weights and heights of various age/gender groups of the population, and for carbohydrates and fats based on percentage of total energy intake (TEI) [4].

A comprehensive examination of the Malaysian adult diet has not been undertaken. In order to develop relevant recommendations for dietary improvement that correspond to the population’s eating patterns, the present study examined the best available evidence regarding dietary intake, specifically energy and macronutrient intake during adulthood (i.e., age 19 to 59 years) and assessed whether intakes adhere to Malaysian RNI guidelines. The objectives were to

1. Describe the following dietary characteristics, based on findings from food balance sheet, national surveys and individual studies, using a systematic literature review and meta-analysis:
  - Malaysian diets in terms of levels of energy and macronutrient (protein, fat, and carbohydrates) intake during adulthood;
  - The proportion of the population whose diets adhere to/exceed/fail to achieve Malaysian Recommended Nutrient Intake (RNI) levels for energy and macronutrients;
  - Sources of macronutrients observed in these studies.
2. Discuss the health implications of current energy and macronutrient intakes in this age group;
3. Provide recommendations to improve intake and prevent development of diet-related chronic disease among Malaysians.

Further studies using improved methods of dietary assessment (i.e., multiple (at least two) 24-h recalls in addition to food frequency or diet history questionnaires, and biomarkers to validate self-reported intake) and data analysis (i.e., estimation of usual intake rather than mean or median intake) on a nationally representative sample are needed in order to develop appropriate recommendations for improvement based on the population’s eating patterns. 

## 2. Materials and Methods 

Search strategy: A search for international and local published studies, reports from international organizations (e.g., World Health Organization (WHO), United Nations Children’s Fund (UNICEF), Association of Southeast Asian Nations (ASEAN), and Food and Agriculture Organization (FAO)) and government institutions, unpublished theses and dissertations that examined diet and food intake among Malaysian subjects was conducted on PubMed and Google. The following search terms were used, Malaysia, diet, food, energy, protein, fat, carbohydrate, nutrition survey, food consumption, nutrient intake. Details are shown in the supplementary table (Table S1).

Ethics approval: Since information was obtained from existing studies and published data, ethics approval was not required.

Inclusion criteria: Criteria for selecting studies and reports were (1) included dietary or nutrient intake of various adult population groups as a variable, (2) covered the period January 2006 to June 2017, (3) done in apparently healthy free-living populations aged 18 years and above, (4) assessed dietary intake in terms of quantity (i.e., consumption of kilocalories of energy, grams carbohydrate, protein, and fat), (5) estimated the amount of energy and protein consumed as a percentage of the Malaysian Recommended Nutrient Intake (RNI) and carbohydrates and fat as percent of total energy intake (TEI). 

Exclusion criteria. The following studies were excluded (1) case studies; (2) studies done on the following types of subjects: institutionalized, disabled subjects, and those with genetic disorders; subjects with chronic disease and medical conditions; pregnant women; and children and adolescents. Studies were also excluded if they did not express intake in terms of %RNI (for energy and protein) and % TEI (for carbohydrates and fat).

Extraction of data. For the literature review, the following information were extracted directly from studies and reports. (1) Amount of macronutrients consumed per day expressed in the following units, energy (kcal), protein (g), fat (g), carbohydrate (g); (2) percentage of the Malaysian RNI (3) for energy and protein, percent of energy consumed from fat, percent of energy consumed from carbohydrate; and (4) dietary sources of carbohydrates, proteins, and fats ingested by subjects.

For the meta-analysis, studies in the review that presented a common summary statistic for energy and macronutrients were selected. Studies with the following summary statistics were analyzed: mean kilocalories per day (for energy), mean grams per day (protein), and mean % TEI (fat and carbohydrate). Statistics presented in the studies were aggregated and weighted by the size of the sex and age strata, when provided. If a specific study contained stratification by age or sex or both, the overall weighted mean and weighted standard deviation were computed based on these strata. The mean values were then used to compare with the Malaysian RNI level for energy and each macronutrient (labeled “Hypothesized” in the Forest Plots). For the purpose of meta-analysis, the hypothesized values were allowed some small variation, thus a nonzero, though very small, standard deviation (SD) was introduced.

Meta-analysis was done via the metacont statement in R package meta. The random-effect model was used in recognition of the diverse kinds of studies pertaining to the same effect. The standardized mean difference (SMD) was used to compute for effect sizes to account for inherent differences among the different studies and to arrive at a “common” measure of difference, given different variances. The inverse-variance method was used in deriving study weights. The random-effect model gave standardized mean differences and 95% confidence intervals. If the 95% CI contains zero, it signifies that the distribution of effects may in fact center at zero and that a significant effect may be absent. Heterogeneity was assessed under the random effects model using the DerSimonian Laird estimator.

Risk of bias assessment. A risk of bias score sheet was developed specifically for the study and made applicable to non-interventional dietary studies. Individual studies were rated by all authors and the average score taken. Criteria were selected to reflect study characteristics that were relevant to nutrition science (specifically dietary assessment). The following criteria were rated: selection bias (sampling method used and representativeness of adults aged 19 to 59 years); performance bias (dietary assessment method used and measurement of usual intake); and reporting bias (excluded or included over/under-reporters and use of free databases vs. those requiring payment). Scores ranged from 6 to 9 (low risk of bias); 10 to 13 (moderate risk of bias); 14 to 16 (high risk of bias). The score sheet is shown in Supplementary Table S6. 

Criteria for a healthy diet. The study used the following criteria to define an acceptable macronutrient intake, consistent with 2017 Malaysian RNI guidelines [4].

- Energy and protein met at least 80% of the Malaysian RNI;
- Total fat should not exceed 30% of TEI;
- Carbohydrate intake should fall within 50 to 65% TEI.

**Figure 1:**
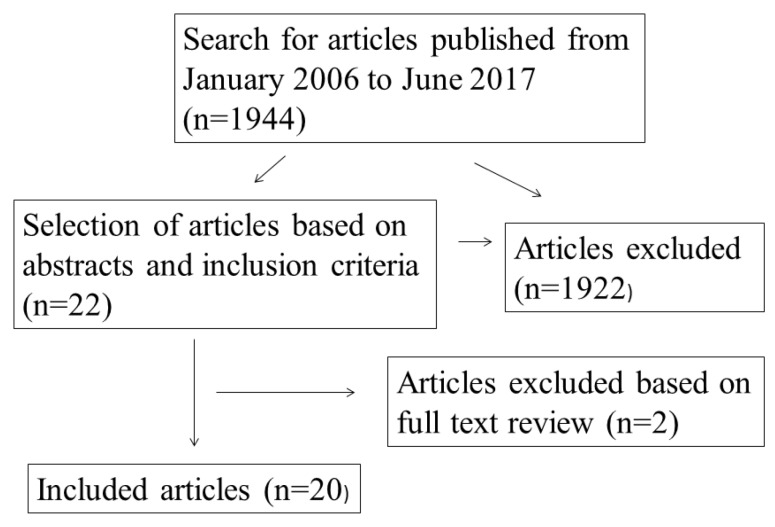
Flow chart for inclusion of studies.

A total of 1944 studies were searched, and 22 studies met the inclusion criteria. Twenty studies were finally selected after vetting through the full articles (Figure 1). All authors agreed on the final selection. Table 1 shows characteristics of included studies. 

## 3. Results

Literature review

Table 2 summarizes the results of studies on energy and macronutrient intake of Malaysian adults and whether they met Malaysian RNI levels. More detailed descriptions of findings from each study are shown in Supplementary Tables S2–S5.

### 3.1. Levels of Energy and Macronutrient Intake

#### 3.1.1. Energy

• Nationwide studies

Food balance sheet data [5] showed that for 2013, the supply of per capita available energy exceeded 100% adequacy. In contrast, the latest 2014 nationwide food consumption survey (Malaysian Adults Nutrition Survey or MANS) [6] showed that adult energy intakes were low and failed to meet at least 80% of the Malaysian RNI. An analysis of 2003 MANS data [8] that excluded over- and under-reporters showed that energy intake was above 100% RNI. Another analysis of MANS 2003 data that included all individuals regardless of reporting status, showed energy intakes were between 62% to 75% RNI [9] (Supplementary Table S2).

- Small studies
  1. Young adults
    Karupaiah et al. [13] showed that women living in urban high rise dwellings aged 19 to 29 years consumed 93% of energy RNI. In contrast, studies on university students [16,19] showed low energy intakes (below 80% RNI).
  2. Older adults
    Three studies [13,15,21] using varied methods showed low energy intake, while two studies [11,24] showed adequate intakes. Women aged 51 to 59 years [13] living in high-rise urban dwellings consumed 78% of energy RNI. Intake of healthy premenopausal Chinese women aged 30 to 45 years was 69% RNI [15]. Women in food secure urban households [21] had low (65% RNI) energy intake. In contrast, women screened for breast cancer showed adequate energy intake (51% met the RNI) [11]. Urban middle-aged Malaysian women showed adequate energy intake (88.5% of the RDA), with postmenopausal women having higher intake (90.3% of RDA) than premenopausal women (87% of RDA) [24].

#### 3.1.2. Protein

• Nationwide studies

Table 2 summarizes the findings on adult protein intake. FAO food balance sheet data [5] showed that per capita protein supply (81 g/day) exceeded individual protein requirements. Both the 2014 and 2003 nationwide surveys [7,9] showed that Malaysian adults tend to consume adequate to high levels of protein (i.e., 80% to over 100% RNI). MANS 2014 [7] showed that median protein intake met 97.7% of RNI, with men having higher intake than women. Almost all sociodemographic groups met at least 80% of the RNI (Supplementary Table S3). The exception was widows and Indian ethnic females who met only 77% and 69% of protein RNI, respectively. In MANS 2003, protein intakes ranged from 92% to 100% RNI [9].

- Small studies
  1. Young adults
    Studies [13,19] showed that most subjects in this age group had adequate protein intake. The exception is one study [16], which showed the majority (68%) of female subjects aged 18 to 24 years did not meet the RNI for protein.
  2. Older adults
    Most studies on older adults showed a tendency towards high protein intake. Intake among women aged 30 to 59 years was 129% of RNI [13]. Regardless of food security status, urban and rural women in Kelantan had protein intakes at 100% RNI or higher [21]. Among pre- and postmenopausal women, intake was 145% and 143% of RDA, respectively [24].

#### 3.1.3. Fat

• Nationwide studies

Table 2 summarizes the findings on fat intake. FAO food balance sheet data [5] showed a fat supply of 89.7 g/capita/day, supplying 28% of available energy. Both nationwide surveys showed adequate to high fat intakes among Malaysian adults. MANS 2014 [7] showed that median fat intake was 46.43 g per day, approximately 29% of median energy intake, and was similar in both men and women. Almost 95% of adults exceeded the RNI [6]. MANS 2003 showed mean fat intake of 46 g/day [9], with men having higher intake than women (Supplementary Table S4).

- Small studies
  Studies in different age groups and using different methods consistently showed adequate to high fat intakes among adults. An examination of the habitual diet of Malaysian Punjabis aged 18 to 59 years showed fat intake was 30% TEI [10].
  1. Young adults
    Studies among university students showed majority either met or exceeded the recommended level [14,16,19]. Young adults with and without acne vulgaris had fat intake above 30% TEI [17].
  2. Older adults
    Two groups of nondiabetic women with a history of gestational diabetes but were currently nondiabetic showed baseline fat intake of 29% and 30% TEI, respectively [12]. Majority of Malay, Chinese, and Indian women screened for breast cancer and found normal showed intake of 25% TEI or higher [11]. Population groups whose fat intakes exceeded 30% TEI were Chinese premenopausal women (36% TEI) [15], male and female Malay subjects (33% TEI) [18], and normal, overweight, and obese women [23]. Rural women with varying levels of food security had higher fat consumption (ranging from 27 to 32% TEI) than urban women (ranging from 24 to 26% TEI) [21]. Among married couples in an urban area in Selangor, consumption of fat was 29% and 30% TEI for husbands and wives, respectively [22]. Healthy men screened for prostate cancer aged 67.8 ± 4.6 years showed adequate fat intake (25% TEI) [20].

#### 3.1.4. Carbohydrates

• Nationwide studies

Table 2 summarizes the findings on carbohydrate intake. FAO food balance data [5] showed that carbohydrate foods supplied approximately 63% of the total per capita energy supply. In MANS 2014 median carbohydrate intake was 195 g/person/day, supplying 55% of median energy supply [7] (Supplementary Table S5). Asma’s [8] analysis of 2003 MANS excluding over- and under-reporters showed carbohydrate intake was 74% of RNI. In contrast, Mahmud et al.’s [6] analysis of MANS 2014 showed that 53.5% of adults had inadequate intakes, 39.1% of males and 41.5% of females met the RNI for carbohydrates, while 6.3% exceeded the RNI.

• Small studies

Nine studies showed that carbohydrate consumption fell within recommended levels, while one study [19] showed low intake. Kaur et al.’s [10] study among Malaysian Punjabis aged 18 to 59 years showed carbohydrate intake was 55% TEI. In young adults, two studies [14,17] showed carbohydrate consumption within recommended levels, while one study [19] showed intake of male and female students below 55% TEI. In older adults, all studies showed adequate to high carbohydrate intakes.

Meta-analysis of selected studies

#### 3.1.5. Energy

**Figure 2:**
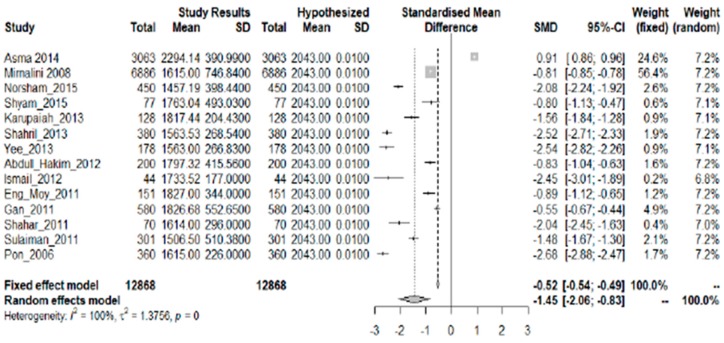
Meta-analysis of studies on energy intake of Malaysian adults.

Fourteen studies with a common summary statistic (mean kcal/day) were included in the analysis (Figure 2). There is statistical evidence that the amount of consumed energy fell below the mean RNI for both sexes in all age groups, shown by the overall SMD of −1.45 (95% CI: −2.06, −0.83). Only the study of Asma [8] showed a significantly higher level of energy intake than the recommended value. While Asma showed an excess of 251 kcal/day, the rest of the studies showed an average deficit of 374 kcal/day. There is a strong descriptive measure of the model’s explanatory success (I^2^ = 100%; *p* = 0.000).

#### 3.1.6. Protein

**Figure 3:**
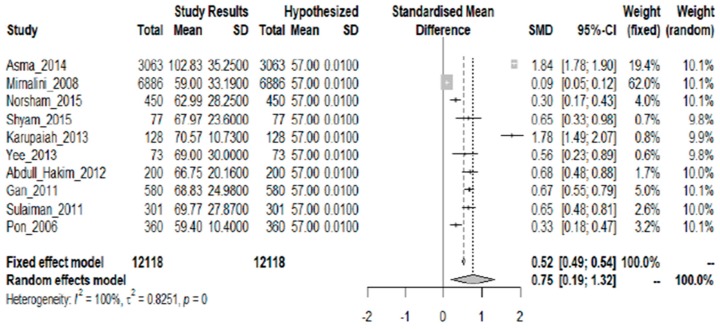
Meta-analysis of studies on protein intake of Malaysian adults.

Ten studies showing protein intake as mean grams/day were included in the analysis. Statistical evidence showed that the required protein levels were met, as shown by the overall SMD of 0.75 (95% CI: 0.19, 1.32) (Figure 3). The average excess over the mean RNI for both sexes in all age groups is 12.71 g. A strong descriptive measure shows the model’s explanatory success (I^2^ = 100%; *p* = 0.000).

#### 3.1.7. Fat

**Figure 4:**
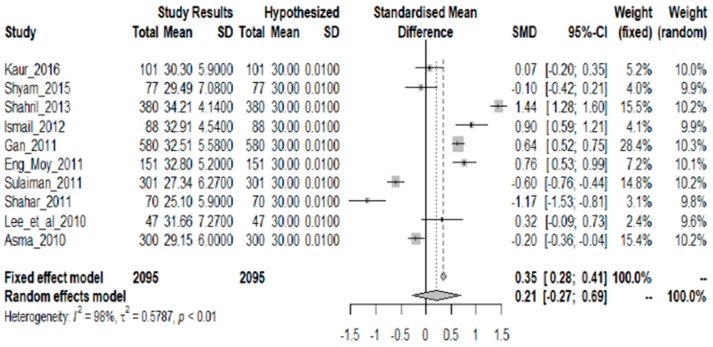
Meta-analysis of studies on fat intake of Malaysian adults.

Ten studies showing fat intake as mean % TEI were included in the analysis. Figure 4 shows that although there is diversity in the results, there is no sufficient statistical evidence that the fat intake is not 30% TEI. Thus it can be asserted that the 30% TEI threshold is upheld as shown by the overall SMD of 0.21 and a 95% CI that contains zero (95% CI: −0.27, 0.69). There is a strong descriptive measure of the model’s explanatory success (I^2^ = 98%; *p* = 0.000).

#### 3.1.8. Carbohydrate

**Figure 5:**
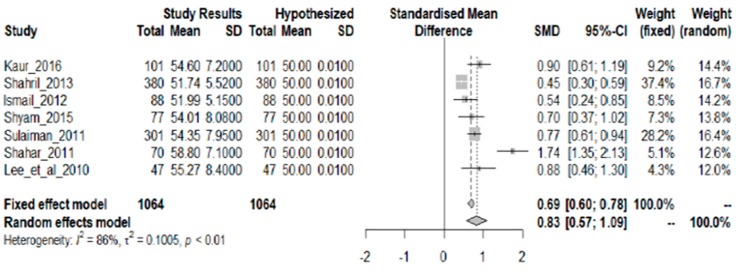
Meta-analysis of studies on carbohydrate intake of Malaysian adults.

Seven studies showing carbohydrate intake as mean % TEI were included in the analysis. Figure 5 shows there is sufficient statistical evidence that the required carbohydrate intake is adequate (more than 50% TEI). This is shown by the overall SMD of 0.83 and a 95% CI (0.57, 1.09). The average excess over the 50% TEI level is 4.39%. There is a strong descriptive measure of the model’s explanatory success (I^2^ = 86%; *p* < 0.01).

Risk of bias assessment. A moderate to high risk of bias was found for the entire set of studies included in the meta-analysis. Individual study ratings using the assessment tool are shown in Supplementary Table S6.

### 3.2. Sources of Macronutrients

Diets are evaluated both in terms of quantity (levels of nutrient intake) and quality (sources of nutrients). This is because at similar levels of intake, physiological effects may vary depending on the source of the nutrient (e.g., saturated vs. unsaturated fat and simple vs. complex carbohydrates). Out of 20 studies in the review, nine identified, to some extent, dietary sources of macronutrients. Supplementary Table S7 summarizes the sources of carbohydrate, protein, and fat in these studies.

#### 3.2.1. Carbohydrate Sources

• Nationwide studies

FAO food balance data [5] showed that cereals, vegetables, and sugar provided the greatest amount (in kg/capita/year) of available carbohydrates in Malaysia. Fruits contributed a lesser amount, and starchy roots provided the least amount. Among cereals, milled rice has the highest amount followed by wheat and wheat products. MANS 2014 [7] showed that carbohydrate-source foods consumed daily by most adults were white rice, sugar (white, brown, Melaka), green leafy vegetables, sweetened condensed milk, and cream crackers. Foods consumed weekly were cabbage, local *kuih* (traditional glutinous rice cakes), noodles, and white bread. The proportion of the population with adequate intake of fruits (≥2 servings) was 9.9%, and that of vegetables (≥3 servings) was 11.2% [7]. 

• Small studies 

White rice was consumed daily by young and older adults. Fruits and vegetables were consumed in lesser amounts, and did not meet Malaysian dietary guidelines of five servings/day. Regardless of food security status, women aged 21 to 49 years consumed more than six servings of grains and cereals daily (consistent with Malaysian dietary guidelines), but less than two servings of fruits and vegetables [21]. 

Studies in young adults [14,19] showed that rice was the most consumed carbohydrate food, followed by vegetables. Fruits were less widely consumed. Shahril et al. [14] found that students consumed less than two servings of rice and vegetables, and less than half a serving of fruit daily. Gan et al. [19] found that while more than 70% of students ate bread, rice, or noodles daily, only 60–62% ate vegetables, and 23–25% ate fruits daily. Abdull Hakim et al. [16] found that less than 50% of male and female students consumed vegetables daily, while less than 20% consumed fruits daily.

In older adults, Asma et al. [22] showed that husbands and wives consumed 97–100% of the recommended servings for grains, but only 68–70% of the recommended servings for vegetables, and 49–60% of recommended servings for fruits. Pon et al. [24] found that while 90% of subjects consumed vegetables and rice daily, only 71% consumed fruits daily. 

#### 3.2.2. Protein Sources

• Nationwide studies

FAO food balance sheet data [5] showed that the country’s protein supply comprised 56% from animal products and 44% from vegetable products. Among animal products, the greatest supply was from fish and seafood (34%) and meat and poultry (33%), followed by milk (15%) and eggs (10%). Among meats, 73% came from poultry and 28% from bovine/pig/mutton/goat meat. Lesser amounts of protein came from pulses, nuts, and seeds (6%). MANS 2014 [7] showed that protein-source foods consumed daily by most adults were marine fish and hen egg, while foods consumed weekly were chicken followed by other types of legumes (long bean, French bean, *kacang botol* (winged bean)).

• Small studies

Sulaiman et al. [21] found that regardless of food security status, subjects aged 21 to 49 years consumed two to three servings of fish, meat, poultry, or legumes daily, but less than one serving of milk and dairy products. Studies among young adults suggested that meat and poultry were consumed more than other sources. Shahril et al. [14] found that students consumed one to two servings of poultry but less than one serving of fish and other sources daily, while legumes were not consumed. Gan et al. [19] showed that 59–71% of male and female students consumed meat or chicken daily, 33–40% consumed fish daily, 21–28% consumed milk daily, and 17–27% consumed legumes daily.

In older individuals, Pon et al. [24] showed that most (36%) consumed fish and seafood, followed by milk (35%), and legumes and products (15%). Poultry and meat were consumed only by a minority of subjects (13.1% and 4.7%, respectively). The authors acknowledged that their sample may not be representative of the population, being from an urban community with higher income and better education.

#### 3.2.3. Fat Sources

• Nationwide studies

The FAO food balance sheet data [5] showed that, of the available fat supply, 62% came from vegetable sources while 38% came from animal sources. Vegetable oils comprised 75% of the vegetable fat supply, and predominant sources were palm oil (41%) and palm kernel oil (26%). Among animal sources, animal fat (saturated fat) provided the greatest amount. Fish liver oil (unsaturated fat) provided 6% of animal fat supply.

• Small studies

Only three studies examined fat sources among subjects. Shahril et al. [14] found that students consumed less than one serving of deep-fried foods daily. In older adults, one study [18] found that saturated fat, monounsaturated fat (MUFA), and polyunsaturated fat (PUFA) comprised 10.1%, 8.5%, and 5.3% of total energy/day, respectively. Another study [22] found lower levels of saturated fat, comprising 4.3% and 4.9% of dietary energy among husbands and wives, respectively. In the latter two studies, food sources of dietary fat were not identified.

## 4. Discussion

The present review, as well as the meta-analysis of selected studies, consistently showed that, in both nationwide and individual studies, there was adequate to high protein and fat intake particularly among older adults, in different groups of subjects regardless of the dietary assessment method used. Findings were inconsistent with respect to carbohydrates. The meta-analysis of purely small studies showed adequate carbohydrate intake, while the 2014 nationwide survey (review data) showed low carbohydrate intake. For energy, both the review and meta-analysis results suggested that current adult diets were low in energy and failed to meet recommended national guidelines. Although information is limited, studies showed that macronutrient sources were animal products (poultry, meat, and fish) for protein, vegetable oils (palm oil and palm kernel oil) for fat, and white rice, vegetables, and sugar for carbohydrates.

### 4.1. Energy Intake and Health

MANS data suggested low and decreasing energy intake among adults. Compared with MANS 2003, median energy intake in 2014 decreased by approximately 771 kcal [25]. Intake among males decreased from 1722 to 1464 kcal, while intake among females increased from 1400 to 1437 kcal [25]. 

Low caloric intake is shown to be beneficial for long-term health. Healthspan is defined as the number of years lived in the best possible health, and combines both concepts of longevity and healthy aging [26]. Factors that have been shown to contribute to healthspan are mild caloric restriction, food quality, genes, and physical activity [27,28]. Among dietary interventions, caloric restriction (CR) without malnutrition is shown to have the greatest health benefits for both humans and animals, including reduced cardiometabolic risk factors and improved immune function [28,29,30,31]. Among aging Malaysian men, caloric restriction for three months resulted in body weight and fat loss, alleviated depression, and improved quality of life [32]. A large community-based cohort study involving 1993 older Malaysian adults showed that not practicing calorie restriction was a risk factor for poor cognitive performance [33].

However, MANS results showing reduced energy intake do not support the increasing trend of overweight and obesity in Malaysia. Baharudin [34] examined the prevalence of overweight and obesity among adults after 10 years. Paradoxically, in spite of low and decreasing energy intake, prevalence of overweight increased from 26.7% in MANS 2003 to 32.4% in MANS 2014, while obesity increased from 12.2% to 18.5%. Among women, overweight increased from 24.8% in 2003 to 31.4% in 2014, while obesity increased from 14.7% to 22.9%. Among men, overweight increased from 28.6% to 33.3%, while obesity increased from 9.7% to 14.5%. Unlike nationwide surveys, findings from individual studies do not go in the same direction. Lee et al. [23] showed that energy intake was positively correlated with BMI among overweight and obese Malay women, as overweight and obese subjects consumed 125 and 434 kcal/day, respectively, more than their normal weight counterparts. Asma’s analysis of 2003 MANS data [8] excluding over- and under-reporters showed that total energy intake increased from 2074 kcal to 2552 kcal/day as BMI classification increased from underweight to obese, respectively. Pon et al.’s [24] study among urban middle-aged women showed that energy intake was the strongest predictor of BMI and waist circumference.

### 4.2. Protein Intake and Health

The present review suggests that protein intake of Malaysian adults, especially older adults, either met or exceeded national recommendations, and that animal rather than vegetable protein was the main source. Findings from cross-sectional studies showed that a “meat, rice, and noodles” diet among Chinese Malaysians was associated with higher BMI, blood glucose, and lipids, with certain alleles further increasing the risk from this dietary pattern [35], and that soy milk consumption reduced risk of obesity [36]. Case–control studies among Malaysian cancer patients suggested that red meat intake was associated with increased risk of colorectal adenoma (CRA) (OR 2.51, 95% CI 1.02, 6.28) [37] and prostate cancer (OR 12.23, 95% CI 3.89, 39.01) [38], while soybean and soy products reduced the risk of CRA (OR 0.38, 95% CI 0.15, 0.98) [37]. 

Wan Shakira et al. [25] reported that the quantity of protein consumed by Malaysian adults increased from 55 kcal (13.7 g protein) in 2003 to 57 kcal (14.2 g protein) in 2014. An evaluation of the extent to which Malaysian adults met Malaysian Food Pyramid guidelines [39] showed that the majority (66.8%) met the daily recommended servings for “meat, poultry, and egg” while most did not meet recommended servings for other protein sources specifically legumes and nuts (82.9%), milk and dairy products (75.6%), fish and fish products (68.7%).

There are no Malaysian longitudinal studies that examined protein intake and disease development. Current findings suggest adverse effects of a high animal protein diet during adulthood. In Levine et al.’s study [40], 6381 subjects aged 50 and above from the U.S. National Health and Nutrition Examination Survey (NHANES) III were followed up after 18 years and showed an association between protein intake (comprising 11% animal protein, 5% plant protein) and mortality from all causes, cardiovascular disease, and diabetes. High protein intake (≥20% calories from protein) was associated with a 5-fold increase in diabetes mortality across all ages. For cancer and all-cause mortality, the association between protein intake and mortality differed for middle-age (50–65 years) and older (66+ years) adults. Respondents aged 50 to 65 reporting high protein intake had a 75% increase in overall mortality and a 4-fold increase in cancer death risk during the following 18 years. When percent calories from animal protein was controlled for, the association between total protein and all-cause or cancer mortality was significantly reduced, suggesting animal proteins were responsible for a significant proportion of these relationships. But in adults over age 65, high protein intake was associated with reduced cancer and overall mortality, compared with those consuming low protein (below 10% of calories) [40]. 

Other studies suggest that reducing the intake of animal protein while increasing that of vegetable protein (legumes, beans, and nuts) contributes towards healthy aging. Willcox et al. [41] characterized the traditional Okinawan diet, a region known for its centenarians. The traditional diet provides moderate protein with an emphasis on vegetable protein (legumes and soybeans), fish, and lean meats as sources. The distribution of energy in the traditional Okinawan diet is 9% protein and 85% carbohydrate, the lowest reported values for dietary percent protein in human populations with an adequate food supply [42]. The protein to carbohydrate ratio in the traditional diet is 1:10, similar to diets that optimize lifespan in animal studies of aging. Studies are needed to determine if levels of animal protein currently consumed by Malaysian adults could adversely affect later health.

### 4.3. Fat Intake and Health

Wan Shakira et al. [25] reported that population fat intake increased slightly from 45.5 (5.1 g fat) in 2003 to 46.4 kcal (5.2 g fat) in 2014. Nationwide and individual studies did not describe dietary sources of fat. One study [18] suggested that saturated fats were more dominant in the Malaysian diet, compared with PUFA and MUFA. FAO data showed that vegetable oils, specifically palm and palm kernel oils, were the dominant fat sources. While none of the included studies described palm oil or palm kernel oil consumption, market reports showed that domestic consumption of palm oil increased from 2685 million metric tons in 2016 to 3117 million metric tons in 2017, a growth rate of 16.09% [43]. During the same period, palm kernel oil consumption increased from 1462 to 1534 million metric tons, a growth rate of 4.92% [44].

In 2016, the principal cause of death in Malaysia was ischemic heart diseases [45]. Risk factors for cardiovascular disease (CVD), based on the 2015 National Health and Morbidity Survey (NHMS), included overweight, obesity, and hypercholesterolemia [46]. The 2015 NHMS showed that 51.2% of the population were either overweight or obese, and 47.7% had hypercholesterolemia [46]. 

Excessive saturated fat consumption leads to hypercholesterolemia, a condition that predisposes to higher CVD risk [47,48]. Palm oil contains triacylglycerides comprising 50% saturated fatty acids (45% palmitic acid and 5% stearic acid), 40% MUFA (oleic acid), and 10% PUFA (linoleic acid). Due to its saturated fat content, palm oil is thought to increase CVD risk [47]. There is an ongoing debate about the effect of palm oil consumption on CVD. A multicountry analysis [49] showed that in 23 developing countries (excluding Malaysia), increases in palm oil consumption were associated with higher mortality due to ischemic heart disease (IHD), even after adjusting for smoking, a major CVD risk factor. For every additional kilogram of palm oil consumed annually per capita, IHD mortality rate increased by 68 deaths per 100,000 (95% CI 21, 115). When per capita consumption of beef, coconut oil, milk, pork, or chicken were each individually included in the regression, the estimated effect of palm oil in these countries remained significant at or above 55 deaths per 100,000. 

However, a systematic review [50] could not establish strong evidence for or against palm oil consumption relating to CVD risk and CVD-specific mortality. Mancini et al. [47] discussed both favorable and unfavorable changes observed in CVD biomarkers when palm oil constitutes the main dietary saturated fat. A favorable effect is reduced atherogenic potential associated with the reduced percentage of palmitic acid occupying the *sn*-2 position in palm oil triacylglycerides (TAG), compared with animal fat. In TAG, higher percentages of palmitic acid (PA) in the *sn*-2 position are related to the most atherogenic profiles, and modifying the TAG structure to reduce the presence of PA in this position reduces atherogenic potential [47]. Comparison of different fats for the percentage of PA in the *sn*-2 position follows, animal butter (66%), human milk (58%), bovine milk (38%), palm oil (4.4%), and olive oil (0.3%) [47]. Other favorable effects of palm oil are its high content of antioxidants, beta-carotene, vitamin E, and other phytonutrients [51,52,53]. When used in the fresh (not oxidized or reheated) state, the benefits of palm oil include reduction in risk of arterial thrombosis and atherosclerosis, inhibition of endogenous cholesterol biosynthesis, platelet aggregation, reduced blood pressure [53], and protection against a number of chronic diseases [51].

Observed negative effects of excess palmitic acid include mitochondrial dysfunction mediated by oxidative stress, known as lipotoxicity [47]. It has been shown that high palm oil levels induce toxicity to the liver [53], leading to a recommendation for its consumption in moderate amounts. Excessive palm oil might trigger changes in gut microbiota components (i.e., the death of Gram-negative microbiota) resulting in increased inflammation [47]. However, among normocholesterolemic subjects consuming the recommended level of fat intake, there is no clear evidence demonstrating an association of palmitic acid consumption and CVD-risk increase [47]. The atherogenic power of palm oil is thought to be low when consumed within balanced diets. Palm oil’s adverse effects could be due to a dose–response relationship [47], or the use of oxidized cooking fat [53]. More rigorous investigations are needed to define the net advantages and disadvantages induced by palm oil consumption on CVD among Malaysians [47].

Aside from palm oil, there are other sources of saturated fat, particularly, animal protein and dairy products. The net effect of a high animal protein intake combined with high palm oil intake might be additive, resulting in high overall saturated fat intake in the Malaysian diet. Studies are needed to determine whether this might account for high levels of overweight/obesity and hypercholesterolemia seen among Malaysian adults. For fat in general, a case–control study by Matalqah et al. [54] found that practicing a low fat diet was strongly protective against breast cancer development among Malaysian women (OR = 0.53). Among Malaysian patients with coronary heart disease, low level of PUFA in blood was a risk factor for depression and acute myocardial infarction [55].

### 4.4. Carbohydrate Intake and Health

MANS data showed that carbohydrate intake decreased from 221 kcal (55 g) in 2003 to 195 kcal (49 g) in 2014 [25]. The latest nationwide survey suggested that Malaysian adults failed to consume the recommended level of carbohydrates [6]. In 2003 and 2014, rice, sugar, and sweetened condensed milk were among the top food items consumed daily [56]. In 2014, 98.6% of adults consumed sugar sweetened beverages (SSB) with mean intake of two glasses daily [57]. Studies suggest that the fructose moiety of sucrose and high fructose corn syrup may contribute to obesity particularly visceral obesity, independent of caloric intake [58,59]. DiNicolantonio et al. [48] discussed evidence linking added sugars to CHD, concluding that sugar is more of a problem than fat in increasing CHD risk. A study [60] on the levels of consumption and sources of added sugar among Malaysians found insufficient evidence regarding consumption, necessitating further studies to elucidate this aspect of carbohydrate intake.

There are no Malaysian longitudinal studies that examined carbohydrate intake and disease development. Case–control studies among Malaysians showed that fruits and vegetables, particularly tomatoes, were associated with reduced risk of prostate cancer [20,38] and colorectal adenoma [37], while a higher healthy eating index score and antioxidants associated with fruit and vegetable intake reduced risk of breast cancer [61,62]. Among Chinese Malaysian adults [36], a perception that “a balanced diet consisted mainly of vegetables” was associated with reduced risk of obesity. Malaysian women with low fiber intake (<10 g/day) had a 2.2 times higher risk of developing breast cancer [63]. Among patients with coronary heart disease, low levels of dietary fiber and carbohydrates were associated with severe depression and increased risk of acute myocardial infarction [55].

Current findings suggest a need for high levels of “healthy” carbohydrates during adulthood. Animal studies using the “Geometric Framework” (GF) approach or “nutritional geometry” [42,64] to determine the effects of macronutrient distribution on health and aging showed that reducing the amount and proportion of dietary protein and increasing the amount of healthy carbohydrates at midlife can delay aging [42]. The GF approach for nutrition was developed by Raubenheimer and colleagues to disentangle the individual and interactive influences of multiple nutrients on the phenotype [65,66]. GF is a state-space modeling approach that explores how an animal responds to the problem of balancing multiple and changing nutrient needs in a multidimensional and variable nutritional environment. Nutrition is considered an n-dimensional space, in which dietary components (protein, carbohydrates, and fats) are represented by separate axes [67]. Responses of individuals (e.g., lifespan and other phenotypic characteristics) to various dietary manipulations are superimposed on this n-dimensional space by plotting response surfaces, and interpreted statistically using General Additives Models (GAM) [67]. Overall, GF studies suggested that a mild calorie-restricted, high carbohydrate (healthy carbohydrates), and low protein diet in adult subjects (below age 65 years) might help people to better cope with age-related dysfunctions and disease, and that low protein high fat diets result in the poorest outcomes [26]. 

### 4.5. Limitations of the Present Review

#### 4.5.1. Sources of Dietary Data 

Food balance sheets provide a comprehensive picture of a country’s food supply during a specified period [68]. The quantities of food available for human consumption, rather than food actually consumed, are presented as per capita supply of calories, protein, and fat for each available food [68]. Nationwide food consumption surveys examine dietary intake of populations by collecting data on actual food and nutrient intake from individuals, using a variety of dietary assessment methods [69]. Data can be used to identify vulnerable groups in the population or differences in consumption by socioeconomic or demographic groups [69]. Nationwide surveys provide more reliable and accurate information on dietary intake of the population than food balance sheets, and therefore useful for health policy guidance. In the absence of nationwide surveys or information therein, individual studies are used as alternative sources of information. However due to small sample sizes, results cannot be readily applied to the general population.

#### 4.5.2. Limitations of Nationwide Food Consumption Survey Methods

• Measurement errors in dietary assessment

Dietary assessment methods to estimate energy intake are imprecise, indirect measures and are prone to measurement error. Measurement error refers to the difference between the value obtained from a measure and the true value of a parameter [70]. For dietary data, measurement error refers to the difference between reported dietary intake over a specified time period and true (i.e., usual or habitual) dietary intake. Some dietary assessment instruments (e.g., food frequency questionnaires) are more prone to error than others (e.g., food weighing and 24-h recalls). If the error is ignored the results may be misleading [70]. 

Twenty four-hour recalls are among the most accurate and least biased methods of assessing diets [70]. However a single recall does not represent a person’s average intake, and multiple recalls are needed to provide a more accurate assessment. For macronutrients, it has been shown that minimum variance may be obtained from not less than two replicate measurements [71]. In the present review, dietary instruments used were (Table 1) multiple (two to three days) 24-h recalls/food records (seven studies), diet history (four studies), FFQ (two studies with one study based on MANS data), and single 24-h recall (six studies with three studies based on MANS data).

• Estimation of mean or median macronutrient intake rather than usual intake

Usual or habitual intake is the focus of nutrition studies because dietary recommendations are intended to be met over time, and diet-health hypotheses are based on dietary intakes over the long term [72]. Usual intake is estimated to determine (1) the proportion of the population at or below a certain level of intake, (2) the relationships between dietary exposures and health outcomes, and (3) the effect of an intervention on dietary outcomes [72]. Collecting a 24-h recall for at least two nonconsecutive days allows application of statistical techniques to estimate usual dietary intake distributions for a group, and the method can be used to determine the proportion above or below a certain threshold [72]. In the present review, all studies estimated macronutrient intake levels either as mean or median (Table 1), and none estimated usual intake.

• Insufficient food composition data

The lack of food composition data for Malaysian foods also contributes to measurement error, as “borrowing” food composition data from other countries may cause incorrect identification of the food being analyzed, leading to biased nutrient intake data resulting in over- or underestimation of the prevalence of inadequate intakes [73]. This type of error is classified as systematic error, and is difficult to quantify [73]. Limitations of Malaysian food composition tables are inadequate number of foods (only 700 types of foods and about 200 cooked foods) dated 1997, and limited number of nutrients. Macronutrients related to obesity and chronic diseases, e.g., type of sugars, dietary fiber, and different fatty acids, are missing. Mixed dishes are estimated using recipes or the most similar (rather than actual) food item. With rapid changes in methods of cooking, food availability, food supply, etc., there is an urgent need for updated food composition data.

• Underreporting of intake among Malaysian adults and reverse causality

Lee et al. [23] assessed accuracy of reporting in a group of overweight and obese Malay women, using identified cut-off points based on the ratio of reported energy intake (EI) and calculated basal metabolic rate (BMR). BMR was calculated using the predictive equation for Malays [74]. Only 41% of subjects were normal energy reporters and the rest were under-reporters. Similarly, an examination of MANS 2003 data found that underreporting occurred in half of the studied population [9]. Obese/overweight individuals may also be consuming (and therefore reporting) less calories (reverse causality), as a healthy eating campaign is being extensively promoted in Malaysia. Other factors are the cross-sectional study design and sampling differences between MANS 2003 and MANS 2014. All these uncertainties indicate that further investigation is needed to confirm whether or not low energy consumption actually exists among Malaysian adults. 

• Non-use of biomarkers to validate self-reported dietary measures 

Dietary biomarkers can objectively assess dietary intake without the bias of self-reported dietary intake errors and overcome the problem of intra-individual diet variability [75]. None were used by any of the studies reviewed, most likely because these methods are expensive. The doubly-labeled water (DLW) method is considered the “gold standard” for measuring free-living energy intake and expenditure [76,77]. Current biomarkers for macronutrients include [75].

- Total fat—PUFA, MUFA, and SFA in plasma and red blood cells
- Added sugar—^13^C in blood glucose; 24-h urinary sucrose and fructose
- Animal protein—24-h urinary nitrogen; ^13^C in hair; ^15^N in hair; 24-h urinary creatinine; 24-h urinary levels of taurine, 1-methylhistidine, 3-methylhistidine

The authors also acknowledge the fact that, due to resource constraints, only free databases were used to find studies, hence it is likely that not all pertinent studies were retrieved.

## 5. Conclusions

The review suggests that Malaysian adults aged 19 to 59 years tend to meet or (more frequently) exceed Malaysian RNI guidelines for protein and fat. This type of diet may have adverse effects on health, but further studies using improved assessment methods are needed to confirm whether this diet is indeed typical among Malaysian adults. Results for energy and carbohydrates are unclear. There is also a mismatch between seemingly low energy intakes that fail to meet RNI guidelines and increasing prevalence of overweight/obesity, while the contribution of added sugar to carbohydrate intake remains to be established. 

Due to methodological limitations, supported by the moderate to high risk of bias found in the studies included in the review, the evidence is inconclusive regarding macronutrient intakes of Malaysian adults and the proportion of the population whose intakes adhere to, exceed, or fail to meet current dietary recommendations. Reasons include limitations in dietary assessment methods used, outdated and limited information in the current food composition database, inappropriate reporting of dietary data (mean, median rather than usual intake), and respondents’ tendency to under-report intake. While nutritional messages encouraging adults aged 19 to 59 years to consume more legumes, beans and nuts, less saturated animal fat and animal protein, and moderate amounts of vegetable fat can be considered, it is not clear whether such messages actually address the problems regarding energy and macronutrient intakes of Malaysian adults. Better information is needed to guide policy decisions and recommendations for dietary improvement.

Future research recommendations include the following,

(1) Use improved dietary assessment methods in national food consumption surveys. Multiple recalls/records (at least 2 nonconsecutive days) in addition to FFQs will provide more accurate dietary data, help reduce under-reporting, and allow estimation of usual or habitual intake.
(2) Conduct randomized intervention trials among Malaysian subjects to determine effects of different types and levels of macronutrient sources on health (e.g., effects of palm oil).
(3) Implement a longitudinal cohort study to establish the association between adult macronutrient intake and development of chronic disease among Malaysians.
(4) In nationwide surveys, use nutrient intake biomarkers to validate self-reported diet measures at least on a subsample. Biomarkers can confirm levels of nutrient intake while self-report measures (i.e., multiple 24-h recalls) can identify food sources of these nutrients.
(5) Update and expand the Malaysian Food Composition Database, for more accurate estimation of energy, macronutrient, and added sugar intake.

## Figures and Tables

**Table 1:** Characteristics of included studies.

Source (year)	Sample Size, Sample Characteristics, Sampling Method	Study Design	Dietary Assessment Method	Study Excluded Over-/Under Reporters of Energy Intake (Yes/No)	Approach to Determine Adequacy of Macronutrient Intake				Macronutrient (Carbohydrate, Protein, and Fat) Food Sources Identified
FAO food balance sheet (2013) [5]	n/a	n/a	Estimate per capita values for the supply of all food commodities (in kg/person/year) and the calories, protein, and fat content	n/a	n/a				Carbohydrate, protein, fat
Nationwide studies					Energy	Protein	Fat	Carbohydrate (CHO)	
Mahmud et al. (2015) [6]	MANS 2014 sample	Secondary analysis of MANS 2014 data	Single 24-h recall	---	Prevalence of adults that met the RNI for energy	Prevalence of adults that met RNI for protein	Prevalence of adults that met RNI for fat	Prevalence of adults that met RNI for CHO	---
MANS 2014 (2014) [7]	2973 adults (1378 men, 1595 women) aged 18 to 59 y (not pregnant or lactating, no specific diet due to illness); multistage stratified cluster sampling	Cross-sectional survey	Single 24-h recall	Yes	Median (kcal/day), % RNI	Median g/day, % RNI	Median g/day, % energy from fat	Median g/day, % energy from CHO	Carbohydrate, protein
Asma (2014) [8]	Secondary analysis of MANS 2003 data; 3063 adults aged 18 to 59 year; multistage stratified cluster sampling	Cross-sectional survey	Semiquantitative FFQ (126 items)	Yes	Mean (kcal/day), % RNI	Mean g/day, % RNI	Mean g/day, % energy from fat	Mean g/day, % energy from CHO	---
Mirnalini et al. (2008) [9]	7349 adults (51% men, 49% women) aged 18 to 59 year; stratified random sampling	Cross-sectional survey	Single 24-h diet recall	No	Mean (kcal/day), % RNI of mean; median; 25th and 75th percentiles	Mean (g/day), % RNI of mean; median; 25th & 75th percentiles	Mean (g/day); median; 25th & 75th percentiles	Mean g/day; median; 25th and 75th percentiles	---
Small studies									
Kaur et al. (2016) [10]	101 adult volunteer Punjabis aged 18 to 59 y from central region of Malaysia; convenience sampling	Cross-sectional	2-day diet record	No	---	---	Median (g/day), % energy from fat	Median (g/day), % energy from CHO	---
Norsham et al. (2015) [11]	450 women aged 30 to 65 year who participated in health screening in Klang Valley, Malays (68%), Chinese (25.1%), Indians (6.1%); convenience sampling	Cross-sectional	Diet history questionnaire (DHQ)	No	Mean (kcal/day), % of subjects meeting/not meeting RNI for energy	Mean (g/day), % of subjects with protein intake 10–35% energy; >35% energy	Mean (g/day), % of subjects with fat intake 25–35% energy; >35% energy	Mean (g/day); % of subjects with CHO intake 55 to 75% energy; >75% energy	---
Shyam et al. (2015) [12]	77 nondiabetic women with previous gestational diabetes, aged 20 to 40 year;	Experimental parallel group design	3-day diet records at baseline	No	----	----	% energy from fat	% energy from CHO	---
Karupaiah et al. (2013) [13]	128 women (45 Malays, 56 Chinese, 27 Indians) aged 19 to 65 year; random selection of clusters and blocks, convenience sample of volunteer households	Cross-sectional	3-day diet records for two weekdays, one weekend	Yes	Mean (kcal/day), % RNI	Mean (g/day), % RNI	---	---	---
Shahril et al. (2013) [14]	380 university students from four public universities in East Coast of Malaysia, aged 18 to 24 year, 47 males, 333 females; generally healthy; random sampling	Experimental parallel group design (only baseline data were used)	Diet history in the last 7 days	Yes	---	---	% energy from fat	% energy from CHO	Carbohydrate, protein, fat
Yee et al. (2013) [15]	73 healthy Chinese premenopausal women aged 30 to 45 year from Klang Valley; convenience sampling	Cross-sectional	Single 24-h recall	No	Mean (kcal/day) vs. RNI = 2180 kcal/day	Mean (g/day) vs. RNI = 55 g/day	Mean (g/day), % energy from fat	Mean (g/day), % energy from CHO	---
Abdull Hakim et al. (2012) [16]	200 adult students from Universiti Teknologi MARA (UiTM), Universiti Putra Malaysia (UPM), Universiti of Selangor (UNISEL), Management and Science University (MSU) aged 18 to 24 year, 90 males, 110 females; not pregnant, bedridden, or having major physical activity problem; convenience sampling	Cross-sectional	Single 24-h recall	No	Mean (kcal/day), % of subjects with intake < RNI, meet RNI, >RNI	Mean (g/day), % of subjects with intake < RNI, meet RNI, >RNI	Mean (g/day), % of subjects with intake < RNI, meet RNI, >RNI	---	Carbohydrates (fruits & vegetables)
Ismail et al. (2012) [17]	88 adults aged 18 to 30 year with and without acne vulgaris attending a tertiary hospital Dermatology Clinic in Kuala Lumpur without chronic disease; purposive sampling	Case control, cross-sectional	3-day food diary (two weekdays, one weekend)	No	---	% energy from protein	% energy from fat	% energy from CHO	---
Eng & Moy (2011) [18]	151 Malay adults, 39 males, 112 females aged 49.9 ± 4.1 year participating in a worksite Wellness Programme in Kuala Lumpur; random sampling	Cross-sectional	3-day diet record (two weekdays, one weekend)	No	---	---	Mean (g/day) for total fat, saturated fat (SFA), & polyunsaturated fat (PUFA), % energy from fat, SFA, PUFA	---	Fat (saturated, monounsaturated (MUFA), PUFA)
Gan et al. (2011) [19]	584 students aged 18 to 24 year from four universities in Klang Valley, 237 males, 347 females; multistage stratified random sampling	Cross-sectional	Two 24-h recalls for 1 weekday, 1 weekend	Yes	Mean (kcal/day), % RNI, % of subjects with intake < RNI & ≥RNI	Mean (g/day), % RNI, % of subjects with energy from protein at <10%, 10–15%, >15%	Mean (g/day), % energy from fat, % of subjects with energy from fat <20%, 20–30%, >30%	Mean (g/day), % energy from CHO, % of subjects with energy from CHO at <55%, 55–70%, >70%	Carbohydrate, protein
Shahar et al. (2011) [20]	70 healthy men aged 40 to 80 year, without past medical history of prostate cancer and not suffering from any unstable chronic diseases, recruited from Kuala Lumpur Hospital and Universiti Kebangsaan Malaysia Medical Centre in Klang Valley; purposive sampling	Case control, cross-sectional	Validated diet history questionnaire	No	---	---	Mean (g/day), % energy from fat	Mean (g/day), % energy from CHO	---
Sulaiman et al. (2011) [21]	301 Malay women aged 21 to 49 year, not pregnant or lactating, living in rural (n = 151) and urban (n = 150) areas in Kelantan; multistage random sampling	Cross-sectional	Single 24-h recall	No	Mean (kcal/day), % RNI	Mean (g/day), % RNI	Mean (g/day), % energy from fat	Mean (g/day), % energy from CHO	Carbohydrate, protein
Asma et al. (2010) [22]	150 married couples from Selangor aged 20 y and above; not practicing any special diet; purposive sampling	Cross-sectional	Two 24-h recalls	No	---	---	Mean (g/day), % energy from fat and saturated fat	---	Carbohydrate, fat (saturated)
Lee et al. (2010) [23]	115 women aged 18 to 59 year working in Klang Valley (six offices of the Employees Provident Fund); purposive sampling	Cross-sectional	Diet history over 7 days	Yes	---	---	Mean (g/day), % energy from fat	Mean (g/day), % energy from CHO	---
Pon et al. (2006) [24]	360 disease-free women aged ≥ 45 year, non-HRT users with intact uterus; living in a suburb in Kuala Lumpur; convenience sampling for a multicenter study	Cross-sectional	Quantitative FFQ	Yes	Mean (kcal/day), % RDA	Mean (g/day), % RDA	---	---	Carbohydrate, protein

n/a—Not applicable; --- No information.

**Table 2:** Summary of study results regarding adherence of Malaysian adults’ energy and macronutrient intakes to national recommendations.

Source	Energy Consumption Level			Macronutrient Consumption Level								
				Protein			Fat			Carbohydrate		
	Met RNI	Below RNI	Above RNI	Met RNI	Below RNI	Above RNI	Met RNI	Below RNI	Above RNI	Met RNI	Below RNI	Above RNI
FAO food balance sheet (2013) [5]	††	††	127% adequacy for 2014–2016	††	††	Available protein supply in 2013 = 81.58 g/capita/day	Per capita fat supply was 28% of energy	††	††	Per capita CHO supply was 63% of energy	††	††
Nationwide studies												
Mahmud et al. (2015) [6]	---	66.5% of adults had energy intakes below the RNI	---	---	---	50.7% of adults had protein intake exceeding the RNI	---	---	94.9% of adults exceeded the RNI for fat	---	53.5% of adults had CHO intake below RNI	---
Asma (2014) analysis of MANS 2003 data [8]	††	††	Mean energy intake was 101% RNI for men and 100.8% RNI for women	††	††	Protein intake was 180.4% RNI for men and 171.2% RNI for women	††	††	Fat intake was 120% RNI for men and 111.8% for women	††	††	CHO intake was 74.4% TEI for men and 74.9% TEI for women
Mirnalini et al. (2008) [9]	††	Energy intake ranged from 66.6% to 75.1% RNI for men and 62.4% to 76% RNI for women	††	Protein intake ranged from 98.4% to 108.1% RNI for men and 92.7% to 103.6% RNI for women	††	††	**	**	**	**	**	**
MANS 2014 (2014) [7]	††	Energy intake for both sexes was 64.4% of RNI	††	Protein intake was 97.7% RNI (both sexes)	††	Protein intake among men was 101.3% RNI	Fat intake was 29% TEI	††	††	CHO intake was 55% TEI	††	††
Small studies												
All ages												
Kaur et al. (2016) [10]	**	**	**	**	**	**	††	††	Fat intake was 30.3% TEI	CHO intake was 54.6% TEI	††	††
Young adults												
Shahril et al. (2013) [14]	**	**	**	**	**	**	††	††	Fat intake was 34.0% TEI in intervention group and 34.4% TEI in control group	CHO intake was 51.9% TEI in intervention group and 51.6% TEI in control group	††	††
Karupaiah et al. (2013) [13]	Energy intake was 93% RNI for age 19 to 29 year and 84% RNI for age 30 to 50 year	††	††	††	††	Protein intake was 129% RNI for age 19 to 50 year	**	**	**	**	**	**
Abdull Hakim et al. (2012) [16]	---	90.9% of males and 72.2% of females had energy intake below RNI	---	---	68% of females had protein intake below RNI	56.4% of males had protein intake above RNI	21.1% of males and 14.6% of females met RNI for fat	---	56.5% of females and 41.5% of males exceeded RNI for fat	††	††	††
Ismail et al. (2012) [17]	**	**	**	**	**	**	††	††	Fat intake ranged from 31.9% to 33.7% RNI	CHO intake ranged from 50.5% to 53.1% TEI	††	††
Gan et al. (2011) [19]	---	73% of males and 80.5% of females had energy intake below RNI	---	---	---	74.7% of males and 54.4% of females had protein intake above RNI	42.6% of males and 45.5% of females had fat intake between 20 to 30% TEI	---	35.9% of males and 27.7% of females had fat intake >30% TEI	---	64.6% of men and 60.6% of women had CHO intake <55% TEI	---
**Older adults**												
Shyam et al. (2015) [12]	**	**	**	**	**	**	Baseline fat intake was 29% TEI in LGI group and 30% TEI in CHDR group	††	††	CHO intake ranged from 53% to 55% TEI	††	††
Norsham et al. (2015) [11]	51% of women met RNI for energy	----	---	---	---	64.4% of normal women and 70.9% of women with breast adiposity had protein intake above RNI	---	---	Fat intake was >35% TEI in 87% of normal women and 79% of women with breast adiposity	---	---	CHO intake was >75% TEI in 82% of normal women and 83.5% of women with breast adiposity
Yee et al. (2013) [15]	††	Mean energy intake fell below the RNI	††	††	††	Mean protein intake exceeded the RNI	††	††	Mean fat intake was 36% TEI	**	**	**
Karupaiah et al. (2013) [13]	††	Energy intake was 78% RNI for age 51 to 59 year	††	††	††	Protein intake was 129% RNI for age 51 to 59 year	**	**	**	**	**	**
Shahar et al. (2011) [20]	**	**	**	**	**	**	Fat intake was 25.1% energy	††	††	CHO intake was 58.8% TEI	---	---
Eng & Moy (2011) [18]	**	**	**	**	**	**	††	††	Fat intake was 32.8% TEI	**	**	**
Sulaiman et al. (2011) [21]	Energy intake was 90.15% RNI among food secure rural respondents	Energy intake ranged from 53.99% to 77.44% RNI among food secure urban and food insecure urban and rural respondents	††	††	††	Protein intake ranged from 100.06% to 154.42% RNI for food secure and food-insecure women in urban and rural areas	Fat intake of food insecure (rural & urban) and food secure urban women ranged from 23.55% to 29.62% TEI	††	Fat intake of food secure rural respondents was 32.38% TEI	CHO intake in food secure and insecure women in rural and urban areas ranged from 50% TEI to 59% TEI	---	---
Lee et al. (2010) [23]	**	**	**	**	**	**	††	††	Fat intake ranged from 31.0% to 33.5% TEI	CHO intake ranged from 54.4% TEI to 55.9% TEI	---	---
Asma et al. (2010) [22]	**	**	**	**	**	**	Fat intake was 28.6% TEI for men and 29.7% TEI for women	††	††	**	**	**
Pon et al. (2006) [24]	Energy intake was 88.5% RDA	††	††	††	††	Protein intake was 144% of RDA	**	**	**	**	**	**

†† No data; --- Less than half of the subjects had this level of intake; ** Not computed by study author/s.

## Supplementary Materials

The following are available online at http://www.mdpi.com/2072-6643/10/11/1584/s1, Table S1: Pubmed search terms and search strategy, Table S2: Findings on energy intake of Malaysian adults, Table S3: Findings on protein intake of Malaysian adults, Table S4: Findings on fat intake of Malaysian adults, Table S5: Findings on carbohydrate intake of Malaysian adults, Table S6: Assessment for risk of bias, Table S7: Sources of macronutrients in included studies.
