# Supplementary material for: Adherence of Malaysian Adults’ Energy and Macronutrient Intakes to National Recommendations: A Review and Meta-Analysis
Source: Nutrients. 2018 Oct 28;10(11):1584. doi: 10.3390/nu10111584 (PMC6266340; doi:10.3390/nu10111584)
Supplement: Supplementary file 1 [file nutrients-10-01584-s001.pdf]

Supplementary Materials

Table S1. Pubmed search terms and search strategy.

Search terms	Search strategy	No. of studies	No. selected
Diet, food, Malaysia	((“diet”[MeSH Terms] OR “diet”[All Fields]) AND (“food”[MeSH Terms] OR “food”[All Fields])) AND (“malaysia”[MeSH Terms] OR “malaysia”[All Fields]) AND (“2007/06/26”[PDat] : “2017/06/22”[PDat] AND “humans”[MeSH Terms])	234	6
Fat, Malaysia	(fat[All Fields] AND (“malaysia”[MeSH Terms] OR “malaysia”[All Fields])) AND “humans”[MeSH Terms]	183	6
Protein, food, Malaysia	((“proteins”[MeSH Terms] OR “proteins”[All Fields] OR “protein”[All Fields]) AND (“food”[MeSH Terms] OR “food”[All Fields])) AND (“malaysia”[MeSH Terms] OR “malaysia”[All Fields]) AND (“2007/07/07”[PDat] : “2017/07/03”[PDat] AND “humans”[MeSH Terms])	283	0
Nutrition survey, Malaysia	((“nutrition surveys”[MeSH Terms] OR (“nutrition”[All Fields] AND “surveys”[All Fields]) OR “nutrition surveys”[All Fields] OR (“nutrition”[All Fields] AND “survey”[All Fields]) OR “nutrition survey”[All Fields]) AND (“malaysia”[MeSH Terms] OR “malaysia”[All Fields])) AND (“2007/07/15”[PDat] : “2017/07/11”[PDat])	181	8
Food consumption, Malaysia	((“food”[MeSH Terms] OR “food”[All Fields]) AND (“economics”[MeSH Terms] OR “economics”[All Fields] OR “consumption”[All Fields]) AND (“malaysia”[MeSH Terms] OR “malaysia”[All Fields])) AND (“2007/07/15”[PDat] : “2017/07/11”[PDat] AND “humans”[MeSH Terms])	192	1
Nutrient intake, Malaysia	((“energy intake”[MeSH Terms] OR (“energy”[All Fields] AND “intake”[All Fields]) OR “energy intake”[All Fields] OR (“nutrient”[All Fields] AND “intake”[All Fields]) OR “nutrient intake”[All Fields]) AND (“malaysia”[MeSH Terms] OR “malaysia”[All Fields])) AND (“2007/07/15”[PDat] : “2017/07/11”[PDat] AND “humans”[MeSH Terms])	105	1
Carbohydrates, Malaysia	(carbohydrate OR monosaccharide OR disaccharide OR polysaccharide), malaysia	766	7
Total		1944	21
FAO food balance sheet			1
Overall total			22

Table S2. Findings on energy intake of Malaysian adults

**Source (y)**	**Sample**	**Method**	**Results**			
FAO food balance sheet (2013)[5]	n/a	Calculated from available food supply	Available total energy supply = 2916 kcal/capita/day<br>Average dietary energy supply adequacy (3-yr average)<br>- 2014-2016 = 127%<br>- 2012-2014 = 124%			
Nationwide studies						
Mahmud et al. (2015)[6]	MANS 2014 sample	Single 24-hr recall	Prevalence of Malaysian adults that met the RNI for energy = 23% (95% CI 20.9, 25.2)<br>- Males = 20.1% (95% CI 17.3, 23.3)<br>- Females = 26.1% (95% CI 23.5, 28.9)<br>10.6% of adults had intakes exceeding the RNI<br>66.5% of adults had intakes below the RNI			
MANS 2014 (2014)[16]	2973 individuals aged 18 to 59 years	Single 24-hr recall	Demographic characteristic	Energy intake/day (% RNI of median)		
				Both sexes	Men	Women
			Malaysia	64.4	66.5	62.1
			Zone			
			- Peninsular Malaysia	63.3	66.7	60.4
			- East Malaysia	65.7	66.4	65.5
			Strata			
			- Urban	65.8	69.6	62.6
			- Rural	62.1	63.4	61.4
			Age group (years)			
			- 18-19	65.5	62.6	71.3
			- 20-29	68.9	67.4	70.8
			- 30-39	65.0	70.2	61.1
			- 40-49	63.7	66.6	59.5
			- 50-59	59.4	61.3	58.7
			Ethnicity			
			- Malay	2864.7	67.4	62.0
			- Chinese	66.8	69.6	64.4
			- Indian	58.3	66.5	52.8

			- Bumiputera Sabah	62.7	63.1	62.3
			- Bumiputera Sarawak	63.8	62.1	65.5
			- Others	64.1	67.0	61.4
			- Other Bumis	55.6	55.5	55.6
			Marital status			
			- Never married	65.2	64.5	65.9
			- Married/cohabiting	64.5	67.4	61.8
			- Divorced/separating	60.4	64.4	59.2
			- Widow	56.0	-	55.9
			Educational level			
			- Non-formal	51.9	51.8	52.8
			- Primary	60.7	64.4	58.4
			- Secondary	64.0	65.8	62.0
			- Tertiary	68.3	69.9	66.0
			- Others	70.9	62.8	74.1
			Work status			
			- Government/semi-government	66.6	71.1	63.1
			- Private	64.8	67.2	62.0
			- Self-employed	64.2	66.5	59.9
			- Unpaid workers	90.0	95.8	90.0
			- Not working	61.1	55.4	61.8
			- Retired	62.7	58.7	69.8
			- students	67.0	63.3	67.7
			Income group			
			- less than RM1500	61.1	63.2	60.2
			- RM1500-RM3500	66.0	69.2	64.7
			- More than RM3500	67.3	70.4	63.3
Asma (2014) analysis of MANS 2003 data[7]	3063 individuals aged 18 to 59 y	126-item FFQ	Age group (y)<br>Men	Energy intake Mean ± SD (kcal/day)	Energy as mean % RNI	
			- 18-29	2463 ± 18.0	---	

			- 30-50	2491 ± 14.1	---
			- 51-60	2408 ± 33.6	---
			- Total for all ages	2472 ± 10.6	101
			Women		
			- 18-29	2083 ± 14.9	---
			- 30-50	2159 ± 13.3	---
			- 51-60	2112 ± 28.1	---
			- Total for all ages	2125 ± 9.4	100.8
			-		
Mirnalini et al. (2008)[8]	6886 adults aged 18 to 59 y	Single 24-hr recall	Age group (y)	Energy intake Mean ± SE (kcal/day)	Energy as mean % RNI
			Men		
			- 18-19	1817 ± 82	74.5
			- 20-29	1805 ± 23	74.0
			- 30-39	1847 ± 24	75.1
			- 40-49	1716 ± 23	69.8
			- 50-59	1638 ± 30	66.6
			Women		
			- 18-19	1419 ± 55	71.0
			- 20-29	1519 ± 22	76.0
			- 30-39	1468 ± 18	67.3
			- 40-49	1387 ± 21	63.6
			- 50-59	1360 ± 28	62.4
			All		
			- 18-19	1621 ± 51	---
			- 20-29	1665 ± 16	---
			- 30-39	1660 ± 16	---
			- 40-49	1555 ± 16	---
			- 50-59	1503 ± 21	---

Small studies

**Author (y)**	**Sample**	**Method**	**Results**		
All ages					
Karupaiah et al. (2013)[9]	128 urban women aged 19 to 65 y	3-day diet records (2 weekdays, 1 weekend)	Age group (y)	Mean energy intake (kcal) ± SD	Energy as % RNI
			19 to 29	1867 ± 175	93
			30 to 50	1838 ± 229	84

			51 to 59	1706 ± 109	78		
Young adults							
Abdull Hakim et al. (2012)[10]	200 students aged 18 to 24 y (45% males, 55% females)	Single 24-hr recall	Subjects	Energy intake (kcal) ± SD	< RNI No. (%)	Meet RNI No. (%)	> RNI No. (%)
			Males - 18 y	1923.09 ± 477.62 (67.7% RNI)	---	---	---
			- 19+ y	1948.25 ± 440.07 (79.8% RNI)	---	---	---
			Total (n=90)	1938.47 ± 452.55	82 (90.9)	---	8 (9.1)
			Females – 18 y	1756.84 ± 363.34 (85.7% RNI)	---	---	---
			- 19+ y	1651.07 ± 388.46 (82.5% RNI)	---	---	---
			Total (n=110)	1681.84 ± 382.72	79 (72.2)	---	31 (27.8)
Gan et al. (2011)[11]	584 students aged 18 to 24 y (41% males; 59% females)	Two 24-h recalls	Subjects	Energy intake (kcal/d) mean ± SD	Energy as % RNI mean±SD	< RNI No. (%)	≥ RNI No. (%)
			Males (n=237)	2120 ± 614	86.9±25.2	173 (73.0)	64 (27.0)
			Females (n=343)	1624 ± 506	81.2±25.3	276 (80.5)	67 (19.5)

Older adults						
Norsham et al. (2015)[14]	450 women aged 30 to 65 y	Diet history questionnaire	Subjects	Mean energy intake (kcal/d) ± SD	Meet RNI No. (%)	Does not meet RNI No. (%)
			Normal	1414±405	152 (34)	140 (31)
			With breast adiposity	1537±386	75 (17)	83 (18)
			Total		227 (51%)	223 (49%)
Yee et al. (2013)[12]	73 healthy Chinese premenopausal women aged 30 to 45y from Klang Valley	Single 24-hr recall	Subjects	Mean energy intake (kcal/d) ± SD	RNI value presented for comparison only (no computations done)	
			All subjects 30 to 45 y	1506 ± 427 (range 455-2938)	RNI = 2180	
Sulaiman et al. (2011)[13]	301 women (151 in rural areas, 150 in urban areas) aged 21 to 49 y (mean age 38.05 ± 7.03 y)	Single 24-h recall; MANS FFQ	Subjects according to food security status	Mean energy (kcal) ± SD	Energy as % RNI (mean ± SD)	
			Rural respondent			
			- Food secure	1959 ± 620.08	90.15 ± 28.54	
			- Moderately insecure	1671 ± 573.18	77.44 ± 26.31	
			- Severely insecure	1602 ± 624.94	73.67 ± 29.93	
			Urban respondent			
			- Food secure	1407 ± 363.90	64.53 ± 16.69	

			- Moderately insecure	1278 ± 379.55	58.63 ± 17.41
			- Severely insecure	1177 ± 443.46	53.99 ± 20.34
Pon et al. (2006)[15]	360 urban middle-aged women aged 51.65 ± 5.4 y	Quantitative FFQ	Subjects	Mean energy (kcal) ± SD	Energy as % RDA
			Females (all)	1615 ± 226	88.5
			Premenopausal	1633 ± 244	87.3
			Postmenopausal	1591 ± 198	90.3

--- No data

Table S3. Findings on protein intake of Malaysian adults

**Source (y)**	**Sample**	**Method**	**Results**
FAO food balance sheet (2013)[5]	n/a	Calculated from available food supply	• Protein supply (g/capita/day) = 81.58<br>• Protein supply (kcal/capita/day) = 326.32; comprises approximately 11% of available energy/capita/day<br>• Average supply of protein of animal origin (3-year average)<br>- 2011-2013 = 44 g/capita/day<br>- 1991-2001 = 42 g/capita/day
Nationwide surveys			
Mahmud et al. (2015)[6]	MANS 2014 sample	Single 24-hr recall	Prevalence of Malaysian adults that met the RNI for protein = 40.4% (95% CI 37.9, 43.0)<br>- Males = 39.4% (95% CI 35.9, 43.0)<br>- Females = 41.5% (95% CI 38.3, 44.8)<br>50.7% of adults had intakes exceeding the RNI<br>8.8% had intakes below the RNI

**Source (y)**	**Sample**	**Method**	Demographic characteristic	Protein intake/day (% RNI of median)		
MANS 2014 [16]	2973 individuals aged 18 to 59 years	Single 24-hr recall		Both sexes	Men	Women
			Malaysia	97.7	101.3	94.9

Zone			
- Peninsular Malaysia	94.8	99.2	90.2
- East Malaysia	102.5	103.2	102.2
Strata			
- Urban	99.4	105.2	95.8
- Rural	95.6	97.2	93.5
Age group (years)			
- 18-19	94.7	101.4	87.3
- 20-29	100.8	101.7	99.5
- 30-39	99.3	106.9	95.7
- 40-49	95.0	98.7	93.3
- 50-59	93.4	96.0	91.6
Ethnicity			
- Malay	95.8	100.7	92.0
- Chinese	110.1	114.8	103.5
- Indian	82.1	89.7	69.2
- Bumiputera	95.7	97.2	92.9
Sabah			
- Bumiputera	99.2	88.4	105.4
Sarawak			
- Others	102.9	106.4	99.6
- Other Bumis	94.9	93.3	94.9
Marital status			
- Never married	97.8	101.1	92.3
- Married/cohabiting	98.6	101.5	96.6
- Divorced/separating	90.9	93.7	87.2
- Widow	77.3	---	77.1
Educational level			
- Non-formal	81.2	80.9	81.7
- Primary	96.1	96.1	96.6
- Secondary	98.1	103.3	91.8
- Tertiary	100.8	101.3	100.2
- Others	99.1	93.4	115.6
Work status			
- Government/semi-government	98.6	100.9	97.8
- Private	97.3	99.1	94.1
- Self-employed	100.4	103.0	93.7
- Unpaid workers	104.8	135.7	104.8

			- Not working	94.5	88.0	95.1
			- Retired	92.7	85.0	112.1
			- students	88.4	96.1	84.0
			Income group			
			- less than RM1500	93.5	96.0	91.3
			- RM1500-RM3500	102.3	106.0	98.1
			- More than RM3500	100.7	106.0	97.8
Asma (2014) analysis of MANS 2003 data[7]	3063 individuals aged 18 to 59 y	126-item FFQ	Age group (y)	Mean protein intake (g/d) ± SD		Protein as % RNI
			Men			
			- 18-29	110 ± 1.5		---
			- 30-50	114 ± 1.5		---
			- 51-60	106 ± 3.2		---
			- Total for all ages	112 ± 1.0		180.4
			Women			
			- 18-29	91 ± 1.1		---
			- 30-50	97 ± 1.2		---
			- 51-60	91 ± 1.9		---
			- Total for all ages	94.1 ± 0.8		171.2
Mirnalini et al. (2008)[8]	MANS 2003 data (6886 individuals aged 18 to 59 y)	Single 24-hr recall				
			Men	Mean (g/day) ± SE		Mean % RNI
			- 18-19	65 ± 3.7		104.8
			- 20-29	64 ± 1.0		103.3
			- 30-39	67 ± 1.1		108.1
			- 40-49	63 ± 1.0		101.6
			- 50-59	61 ± 1.3		98.4
			Women			
			- 18-19	53 ± 2.6		96.4
			- 20-29	57 ± 1.0		103.6

			- 30-39	55 ± 0.9	100.0
			- 40-49	53 ± 1.0	96.4
			- 50-59	51 ± 1.3	92.7
			All		
			- 18-19	59 ± 2.3	---
			- 20-29	61 ± 0.7	---
			- 30-39	61 ± 0.7	---
			- 40-49	58 ± 0.7	---
			- 50-59	56 ± 0.9	---
			-		

Small studies

Source (y)	Sample	Method	Results				
All ages							
Karupaiah et al. (2013)[9]	128 urban women aged 19 to 65 y	3-day diet records (2 weekdays, 1 weekend)	Age group (y)	Mean protein intake (g/d) ± SD	Protein as % RNI		
			19 to 29	71 ± 9	129		
			30 to 50	71 ± 11	129		
			51 to 59	71 ± 11	129		
Young adults							
Abdull Hakim et al. (2012)[10]	200 students aged 18 to 24 y (45% males, 55% females)	Single 24-hr recall	Subjects	Mean protein intake (g/d) ±SD	<RNI No. (%)	Meet RNI No.(%)	>RNI No.(%)
			Males - 18 y	67.74 ± 21.22	---	---	---
			- 19+ y	73.62 ± 19.15	---	---	---

			Total	71.33 ± 20.07	38 (42)	2 (2.4)	50 (56.4)
			Females – 18 y	61.59 ± 19.73	---	---	---
			- 19+ y	63.59 ± 20.55	---	---	---
			Total	63.01 ± 20.24	75 (68)	2 (1.9)	33 (30.2)
Gan et al. (2011)[11]	584 students aged 18 to 24 y (41% males; 59% females)	Two 24-h recalls	Subjects	Mean protein intake (g/d) ± SD	Protein as % RNI mean±SD	<RNI No. (%)	≥RNI No. (%)
			Males	80.6 ± 27.1	128.6±43.2	60(25.3)	177(74.7)
			Females	60.7 ± 23.4	110.6±42.6	156(45.5)	187(54.4)
Older adults							
Norsham et al. (2015)[14]	450 women aged 30 to 65 y		Subjects	Mean protein intake (g/day)	Protein intake 10-35% energy No. (%)	High protein intake >35% energy No. (%)	
			Normal (n=292)	59.2±21.3	104 (69.3)	188 (62.7)	
			With breast adiposity (n=158)	70.0±37.9	46 (30.7)	112 (37.3)	
Yee et al. (2013)[12]	73 healthy Chinese premenopausal women aged 30 to	Single 24-hr recall	All subjects 30 to 45 y	Mean protein intake	RNI for study population		

	45 y from Klang Valley			(g/d) ± SD	
				69 ± 30 (range 15-175)	RNI = 55 g/day (presented for comparison only; no computation done by authors)
Sulaiman et al. (2011)[13]	301 women (151 in rural areas, 150 in urban areas) aged 21 to 49 y (mean age 38.05 ± 7.03 y)	Single 24-h recall; MANS FFQ	Subjects according to food security status<br>Rural respondent	Mean protein intake (g/d) ± SD	Protein as % RNI (mean ± SD)
			- Food secure	85.76 ± 29.06	154.42 ± 54.23
			- Moderately insecure	82.09 ± 35.78	148.45 ± 65.49
			- Severely insecure	73.32 ± 29.21	134.47 ± 53.61
			Urban respondent		
			- Food secure	64.37 ± 22.47	117.03 ± 40.05
			- Moderately insecure	59.29 ± 24.68	107.80 ± 44.87
			- Severely insecure	55.03 ± 23.44	100.06 ± 42.61
Pon et al. (2006)[15]	360 urban middle-aged women	Quantitative FFQ	Subjects	Mean protein intake	Protein as % RDA

	aged 51.65 ± 5.4 y			(g/day) ± SD	
			Females (all)	59.4 ± 10.4	144.8
			Premenopausal	59.8 ± 10.9	145.9
			Postmenopausal	58.7 ± 9.5	143.2

--- No data

Table S4. Findings on fat intake of Malaysian adults

**Source**	**Sample**	**Method**	**Results**		
FAO food balance sheet (2013)[5]	n/a	Calculated from available food supply	Fat supply (g/capita/day) = 89.74<br>Fat supply (kcal/capita/day) = 807.66; comprises approximately 28% of available energy/capita/day		
Nationwide studies					
Mahmud et al. (2015)[6]	MANS 2014 sample	Single 24-hr recall	Prevalence of Malaysian adults that met the RNI for fat = 3.4% (95% CI 2.7, 4.4)<br>- Males = 3.1% (95% CI 2.2, 4.5)<br>- Females = 3.7% (95% CI 2.7, 5.1)<br>94.9% of adults had intakes exceeding the RNI<br>1.7% of adults had intakes below the RNI		
MANS 2014[16]	2973 individuals aged 18 to 59 years	Single 24-hr recall	Median fat intake (both sexes) = 46.43 g/day<br>Median percentage of total energy contributed by fat = 29%; similar in men and women		
Asma (2014) analysis of MANS 2003[7]	3063 individuals aged 18 to 59 y	126-item FFQ	Age group (y)	Mean fat intake (g/day) ± SD	Fat as mean % RNI
			Men (RNI 54-82 g; 20-30% TEI)		
			- 18-29	65 ± 1.9	---
			- 30-50	67 ± 2.1	---
			- 51-60	58 ± 4.4	---

			- Total for all ages	65 ± 1.4	120.7
			Women (RNI 46-70 g; 20-30% TEI)		
			- 18-29	52 ± 1.4	---
			- 30-50	53 ± 1.5	---
			- 51-60	42 ± 1.5	---
			- Total for all ages	51.4 ± 0.9	111.8
Mirnalini et al. (2008)[8]	MANS 2003 sample; 6886 individuals aged 18 to 59 y	Single 24-hr recall	Median intake for both sexes (all age groups) = 46 g/day		
			Men	Mean fat intake (g/day) ± SE	Mean as % TEI
			- 18-19	56 ± 3.7	---
			- 20-29	56 ± 0.9	---
			- 30-39	56 ± 1.0	---
			- 40-49	53 ± 1.1	---
			- 50-59	49 ±1.4	---
			Women		
			- 18-19	46 ± 1.9	---
			- 20-29	48 ± 0.9	---
			- 30-39	45 ± 0.8	---
			- 40-49	43 ± 1.0	---
			- 50-59	41 ± 1.2	---
			All		
			- 18-19	51 ± 2.1	---
			- 20-29	52 ± 0.7	---
			- 30-39	51 ± 0.6	---
			- 40-49	48 ± 0.7	---
			- 50-59	45 ± 1.0	---

Small studies

**Source (y)**	**Sample**	**Method**	**Results**

All ages

Kaur et al. (2016)[17]	101 Malaysian Punjabis aged 18 to 59 y	2-day diet records	Subjects	Median fat intake g/day, (P_5, P_{95})	Fat as % energy mean±SD		
			All (both sexes)	61.48 (34.1, 93.2)	30.3±5.9		
Young adults							
Shahril et al. (2013)[18]	380 students aged 18 to 24 y	Diet history for the last 7 days, FFQ	Subjects	Mean fat intake (g/day) at baseline	Fat as % energy		
			- Intervention group	---	34.0±0.3		
			- Control group	---	34.4±0.3		
Abdull Hakim et al. (2012)[10]	n=200 students aged 18 to 24 y (45% males, 55% females)	Single 24-hr recall	Subjects	Mean fat intake (g/d) ± SD	<RNI No. (%)	Meet RNI No. (%)	>RNI No. (%)
			Males - 18 y	91.11 ± 41.84	---	---	---
			- 19+ y	78.36 ± 25.59	---	---	---
			Total	83.32 ± 33.25	19 (21.1)	34 (37.5)	37 (41.5)
			Females – 18 y	79.19 ± 26.31	---	---	---
			- 19+ y	72.04 ± 26.12	---	---	---
			Total	74.12 ± 26.26	16 (14.6)	32 (28.9)	62 (56.5)

Ismail et al. (2012)[19]	88 adults aged 18 to 30 y with and without acne vulgaris	3 day food diaries	Subjects	Fat intake	Fat as % of energy (Mean ± SD)		
			Males				
			- Cases	---	33.3 ± 4.1		
			- Controls	---	33.7 ± 4.0		
			Females				
			- Cases	---	33.3 ± 5.5		
			- Controls	---	31.9 ± 3.9		
Gan et al. (2011)[11]	n=584 students aged 18 to 24 y (41% males; 59% females)	Two 24-h recalls	Subjects	Mean fat intake (g/d) ± SD	Fat <20% energy No. (%)	20-30% energy No. (%)	Fat >30% energy No. (%)
			Males	75.7 ± 26.1	51 (21.5)	101 (42.6)	85 (35.9)
			Females	59.2 ± 21.2	92 (26.8)	156 (45.5)	95 (27.7)
Older adults							
Shyam et al. (2015)[20]	77 nondiabetic women with previous gestational diabetes aged 20 to 40 y with mean age 30.5 ± 9 y	3-day dietary records at baseline	Subjects according to treatment group assignment	Mean fat intake (g/day) ± SD	Fat as % energy (mean ± SD)		
			- LGI (low glycemic index)	62 ± 23	29 ± 8		
			- CHDR (conventional healthy diet recommendations)	60 ± 20	30 ± 6		

Norsham et al. (2015)[14]	450 women (Malaysian, Chinese, Indian) aged 30 to 60 without breast cancer	Diet history questionnaire	Subjects according to breast adiposity	Mean fat intake (g/d) ± SD	Fat intake 25-35% energy No. (%)	High fat intake >35% energy No. (%)
			- Normal breast density	58.3 ± 22.5	38 (53.5)	254 (67.0)
			- Adiposity of the breast	63.5 ± 21.7	33 (46.5)	125 (33.0)
Yee et al. (2013)[12]	73 healthy Chinese premenopausal women aged 30 to 45 y from Klang Valley	Single 24-hr recall	Subjects	Mean fat intake (g/day) ± SD	Fat as % energy	
			All subjects 30 to 45 y	61 ± 23	36	
Eng & Moy (2011)[21]	151 Malay adults (37.6% males, 62.4% females) mean age 49.8 ± 4.1 y	3-day dietary records (2 weekdays, 1 weekend)	Subjects	Mean fat intake (g/d) ± SD	Fat as % energy	
			All (both sexes)	Total fat = 66.8 ± 16.9	32.8 ± 5.2	
				Saturated fat = 22.7 ± 7.04	% energy from saturated fat = 10.1 ± 2.91	
				MUFA = 19.1 ± 5.51	% energy from MUFA = 8.52 ± 2.27	
				PUFA = 12.0 ± 4.46	% energy from PUFA = 5.34 ± 1.97	

Sulaiman et al. (2011)[13]	301 women (151 in rural areas, 150 in urban areas) aged 21 to 49 y (mean age 38.05 ± 7.03 y)	Single 24-h recall; MANS FFQ	Subjects according to food security status	Mean fat intake (g/day) ± SD	Fat as % energy (mean ± SD)	
			Rural respondent			
			- Food secure	69.85 ± 27.96	32.28 ± 4.98	
			- Moderately insecure	57.34 ± 27.29	29.62 ± 6.73	
			- Severely insecure	50.89 ± 24.84	27.97 ± 6.70	
			Urban respondent			
			- Food secure	42.24 ± 14.16	26.88 ± 5.92	
			- Moderately insecure	34.50 ± 16.69	23.55 ± 6.49	
			- Severely insecure	32.81 ± 17.47	24.39 ± 6.11	
Shahar et al. (2011)[24]	70 healthy men aged 40 to 80 y (mean = 67.8 y)	Diet history questionnaire (DHQ)	Mean fat intake ± SD (g/day)		Fat as % energy (mean ± SD)	
			45.0 ± 13.1		25.1 ± 5.9 %	
Lee et al. (2010)[22]	115 women aged 18 to 59 y (mean age 37.2 ± 7.6 y)	Diet history over 7 days	Subjects	---	Fat as % energy (mean ± SD)	
			- normal weight	---	31.0 ± 6.4	
			- overweight	---	32.4 ± 6.6	
			- obese	---	33.5 ± 5.8	
Asma et al. (2010)[23]	150 married couples aged 20 and above (mean age husbands	2-day 24-hr recall, FFQ	Subjects	---	Total fat as % energy (mean ± SD)	Saturated fat as % energy (mean ± SD)

	43.33 ± 11.16 y; wives 41.28 ± 10.93 y)					
			- husbands	---	28.6 ± 5.8	4.3 ± 2.8
			- wives	---	29.7 ± 6.2	4.9 ± 3.1

--- No data

Table S5. Findings on carbohydrate intake of Malaysian adults

**Source (y)**	**Sample**	**Method**	**Results**		
FAO food balance sheet (2013)[5]	n/a	Per capita values calculated from available food supply	Supply for carbohydrate-source foods	Available kcal/capita/day	Estimated % of per capita available energy *
			- Cereals excluding beverages	1279	44 %
			- Sugar & sweeteners	411	14 %
			- Fruits excluding wine	57	2%
			- Vegetables	56	2%
			- Starchy roots	31	1%
			Total	1834	63%
Nationwide studies					
Mahmud et al. (2015)[6]	MANS 2014 sample	Single 24-hr recall	Prevalence of adults that met the RNI for carbohydrate<br>- Males = 39.1% (95% CI 35.6, 42.7)<br>- Females = 41.5% (95% CI 38.3, 44.7)<br>6.3% of adults had intakes exceeding the RNI<br>53.5% of adults had intakes below the RNI		
MANS 2014[16]	4000 adults aged 18 to 59 y	Single 24-hr recall	Median daily intake of carbohydrate was 195 g; median % of energy from carbohydrate was 55%		
Mirnalini et al.	MANS 2003 sample	Single 24-hr recall	Mean CHO intake (both sexes, all age groups) = 232 ± 1.4 g/day; contributed 59% of energy intake<br>Median CHO intake = 221 g/day		

(2008) [8]					
Asma (2014) analysis of MANS 2003[7]	3063 individuals aged 18 to 59 y	126-item FFQ	Age group (y)	Mean CHO intake (g/day) ± SD	Mean %RNI (55-70% TEI)
			Men		
			- 18-29	461 ± 4.2	---
			- 30-50	463 ± 3.3	---
			- 51-60	447 ± 7.5	---
			- Total (all ages)	460 ± 2.5	74.4
			Women		
			- 18-29	393 ± 3.5	---
			- 30-50	403 ± 3.1	---
			- 51-60	391 ± 7.2	---
			- Total (all ages)	397.5 ± 2.2	74.9

Small studies

**Source (y)**	**Sample**	**Method**	**Results**		
All ages					
Kaur et al. (2016)[17]	100 Malaysian Punjabis aged 18 to 59 y (mean age 31.4 ± 11 y)	3-day 24 h recalls	Median CHO intake (g/day)	% energy from CHO Mean ±SD	
			243.9	54.6 ±7.2	
Young adults					
Shahril et al. (2013)[18]	380 students aged 18 to 24 y	Diet history for the last 7 days, FFQ	Treatment group	Mean CHO intake (g/day) ± SD	% energy from CHO Mean ±SD
			Intervention	---	51.9 ± 0.4

			Control		---		51.6 ± 0.4
Gan et al. (2011)[11]	n=584 students aged 18 to 24 y (41% males; 59% females)	Two 24-h recalls	Subjects	Mean CHO intake ± SD (g/day)	CHO intake <55% energy No. (%)	55-70% energy No. (%)	>70% energy No. (%)
			Male	280 ± 91	153 (64.6)	80 (33.8)	4 (1.7)
			Female	214 ± 73	208 (60.6)	135 (39.4)	0 (0.0)
Ismail et al. (2012)[19]	88 adults aged 18 to 30 y	3 day food diaries	Subjects		Mean CHO intake (g/day) ± SD		% energy from CHO Mean ±SD
			Males				
			- Cases with acne		---		50.5 ± 3.6
			- Controls		---		51.7 ± 4.7
			Females				
			- Cases with acne		---		51.8 ± 6.6
			- Controls		---		53.1 ± 4.3
Older adults							
Shyam et al. (2015)[20]	77 nondiabetic women with previous GDM aged 20 to 40 y receiving conventional dietary recommendations (CHDR) or	3-day dietary records at baseline	Treatment group		Mean CHO intake (g/day) ± SD		% energy from CHO Mean ±SD

	low GI education (LGI)					
			- CHDR	245 ± 75		55 ± 9
			- LGI	225 ± 68		53 ± 7
Norsham et al. (2015)[14]	450 women aged 30 to 65 y	Diet history questionnaire	Subjects	Mean CHO intake ±SD (g/day)	CHO intake 55-75% of energy No. (%)	High CHO intake >75% energy No. (%)
			- Normal	162.7±46.5	52 (66.7)	240 (64.5)
			- With breast adiposity	171.3±55.9	26 (33.3)	132 (35.5)
Sulaiman et al. (2011)[13]	301 women (151 in rural areas, 150 in urban areas) aged 21 to 49 y (mean age 38.05 ± 7.03 y)	Single 24-h recall; MANS FFQ	Respondents	Mean CHO intake (g/day) ± SD		% energy from CHO Mean ±SD
			Rural			
			- Food secure	---		50 ± 5.3
			- Moderate food insecurity	---		51 ± 8.8
			- Severe food insecurity	---		53 ± 8.3
			Urban			
			- Food secure	---		56 ± 8.2
			- Moderate food insecurity	---		59 ± 7.4
			- Severe food insecurity	---		57 ± 8.3
Shahar et al. (2011)[24]	70 healthy men aged 67.8 ±4.6 y	Diet history questionnaire	Mean CHO intake ± SD (g/day)			CHO as % TEI (mean ±SD)

			238 ± 54		58.8 ± 7.1%
Lee et al. (2010)[22]	47 normal energy reporters, Malay women aged 20 to 59 y (mean age 37.2±7.6 y)	Food history over a period of 7 days	Weight status	Mean CHO intake (g/1000 kcal) ± SD	% energy from CHO Mean ± SD
			- Normal	138 ± 17	55.1 ± 6.6
			- Overweight	141 ± 23	55.9 ± 9.4
			- Obese	136 ± 21	54.4 ± 8.4

--- No data

* Calculated from food balance sheet data

Table S6. Assessment for risk of bias

Study	Selection bias		Performance bias		Reporting bias		Total score	Classification of risk of bias
	Sampling method	Representative of age 19 to 59 y	Dietary assessment method used	Usual intake measured	Included/excluded over/underreporters	Use of free vs. all including payment-requiring database		
Norsham et al. 2015	2	1	4	2	2	2	13	moderate
Shyam et al. 2015	2	2	4	2	2	2	14	high
Karupaiah et al. 2013	1	1	4	2	2	2	12	moderate
Shahril et al. 2013	1	2	4	2	2	2	13	moderate

Yee et al. 2013	2	2	5	2	2	2	15	high
Abdull Hakim et al. 2012	2	2	5	2	2	2	15	high
Ismail et al. 2012	2	2	3	2	2	2	13	moderate
Eng & Moy 2011	2	2	4	2	2	2	14	high
Gan et al. 2011	1	2	2	2	2	2	11	moderate
Shahar et al. 2011	2	2	4	2	2	2	14	high
Sulaiman et al. 2011	1	2	5	2	2	2	14	high
Asma et al. 2010	2	2	2	2	2	2	12	moderate
Lee et al. 2010	2	1	4	2	1	2	12	moderate
Pon et al. 2006	2	2	6	2	2	2	16	high
							Over all score = 13.352	MODERATE TO HIGH

Scoring guide:

Selection bias indicators	Score
- Sampling method	1 = random sampling; 2 = purposive or convenience
- Representative of adults aged 19 to 59 y	1 = yes; 2 = no, specific groups only
Performance bias indicators	
- Dietary assessment method used	1 = food weighing

	2 = multiple 24-h recalls<br>3 = food diary (multiple days)<br>4 = diet history<br>5 = single 24-h recall<br>6 = food frequency questionnaire (FFQ)
- Usual intake measured	1 = yes; 2 = no
Reporting bias indicator	
- Included over/underreporters	1 = no; 2= yes
- Use of free databases only vs. all databases	1 = all databases used; 2 = only free databases used

Total score classification: 6-9 (low risk of bias); 10-13 (moderate risk of bias); 14-16 (high risk of bias)

Table S7. Sources of macronutrients in included studies

	**Sources of macronutrients**		
**Author (y)**	**Carbohydrates (CHO)**	**Protein**	**Fat**
FAO food balance sheet (2013)[5]	Available supply of CHO sources; kg/capita/day (% of total CHO supply*)	% protein supply from<br>- Vegetal products = 44% (36.19 g/capita/day)<br>- Animal products = 56% (45.4 g/capita/day)	% fat from<br>- Vegetal products = 62% (55.8 g/capita/day)<br>- Animal products = 38% (33.86 g/capita/day)
	- Cereals excluding beverages = 150.12 (46%)	Animal protein sources; kg/capita/day (% of total protein supply*)	Vegetable fat sources; Kg/capita/day (% of veg. fat supply*)
	- Vegetables = 70.43 (22%)	- Fish, seafood = 58.97 (34%)	- Vegetable oils = 16.63 (75% of veg. fat supply)
	- Fruits excluding wine = 45.29 (14%)	- Meat & poultry = 56.25 (33%)	• palm oil = 6.89 (41% of veg. oil)
	- Sugar & sweeteners = 44.12 (14%)	• Meat (bovine, pig, mutton & goat) = 15.22 kg (28% of meat supply)	• palm kernel oil = 4.37 (26%)
	- Starchy roots = 14.91 (5%)	• Poultry = 41.04 kg (73% of meat supply)	• soyabean oil = 2.35 (14%)
	Available cereals (kg/capita/day)	- Milk = 25.28 (15%)	• Coconut oil = 1.13 (7%)
	- Rice, milled = 81.25	- Eggs = 16.57 (10%)	• oilcrops, other = 0.97 (6%)

<table>
<tr><th></th><th></th><th></th><th></th></tr>
<tr>
<td></td>
<td>- Wheat & wheat products = 51.04
- Maize & products = 15.82
- Cereals, other = 1.09
- Oats = 0.87</td>
<td>- Pulses, nuts, seeds = 10.04 (6%)
- Offal edible = 4.03 (2%)</td>
<td>• Maize germ oil = 0.33 (2%)
• Sesame oil = 0.32 (2%)
• Ground nut oil = 0.18 (1%)
- Oilcrops = 5.49 (25% of veg. fat supply)
Animal fat sources (visible fat supply)
<table>
<tr><td>Saturated fat</td><td>Kg/capita/day (% of animal fat supply*)</td></tr>
<tr><td>- Fat, animal raw</td><td>0.69 (63%)</td></tr>
<tr><td>- Butter, ghee</td><td>0.32 (29%)</td></tr>
<tr><td>- Cream</td><td>0.02 (2%)</td></tr>
<tr><td>Total saturated fat</td><td>1.03 (94%)</td></tr>
<tr><td>Unsaturated fat</td><td></td></tr>
<tr><td>- Fish liver oil</td><td>0.07 (6%)</td></tr>
</table>
</td>
</tr>
<tr><td>Nation wide studies</td><td></td><td></td><td></td></tr>
<tr>
<td>MANS 2014[16]</td>
<td>Top carbohydrate-source foods consumed daily by adults and % consuming
- White rice = 89.8%
- Sugar (white, brown, Melaka) = 55.9%
- Leafy green vegetables = 43.2%
- Condensed milk = 23.5%</td>
<td>Top protein-source foods consumed daily by adults (% consuming)
- Marine fish (29.4%)
- Hen egg (14.2%)</td>
<td>NR</td>
</tr>
</table>

	- Cream crackers = 12.9%<br>-		
	Top carbohydrate-source foods consumed weekly by adults and % consuming<br>- Cabbage = 55.7%<br>- Local kuih = 53.9%<br>- White bread = 50.9%<br>- Leafy green vegetables = 47.3%<br>- Noodles = 46.7%	Top protein-source foods consumed weekly by adults (% consuming)<br>- Hen egg = 70.6%<br>- Chicken = 66.9%<br>- Marine fish = 56.4%<br>- Other types of legumes (long bean, French bean, kacang botol) = 47.6%	NR
Small studies			
All ages			
Sulaiman et al. (2011)[13]	No. of servings per day for carbohydrate-source food groups by food security status	No. of servings per day for protein-source food groups by food security status	NR

	Grains & cereals (no. of svgs/day)	Fruits & vegetables (no. of svgs/day)		Meat, fish, poultry & legumes (no. of svgs/day)	Milk & dairy products (no. of svgs/day)
Food-secure	6.44	1.53	Food-secure	2.83	0.52
Moderate insecurity	6.42	1.62	Moderate insecurity	3.08	0.29
Severe insecurity	6.38	1.14	Severe insecurity	2.34	0.23

Young adults

Shahril et al. (2013)[18]	Mean daily servings of carbohydrate-source food groups<br>- Rice = 1.7<br>- Vegetables = 1.4<br>- Beverages with sweetened condensed milk = 1.0<br>- Bread = 0.8<br>- Biscuits = 0.6<br>- Fruits & 100% fruit juice = 0.4<br>- Beverages with added sugar = 0.4<br>- Noodles = 0.3<br>- Sweet dessert = 0.2<br>- Cereals = 0.1	Mean daily servings of protein-source food groups<br>- Poultry = 1.6<br>- Fish = 0.2<br>- Meat = 0.2<br>- Milk = 0.1<br>- Dairy products = 0.1<br>- Egg = 0.1<br>- Nuts & legumes = 0.0	Mean daily servings of fat-source foods<br>- Deep-fried foods = 0.2
Abdull Hakim et al. (2012)[10]	% of students who ate vegetables daily = 43% of males, 42% of females<br>% of students who ate fruits daily = 14% of males, 17% of females	NR	NR
Gan et al. (2011)[11]	Daily intake of carbohydrate-source foods (% of subjects)	Daily intake of protein-source foods (% of subjects)	NR

	Males (%)	Females (%)		Males (%)	Females (%)
Bread/rice/noodles	72.5	70.9	Meat/chicken	70.9	59.4
Vegetables	61.7	60.8	Fish	40.1	33.8
Fruits	24.9	23.1	Milk	27.3	21.6
100% fruit juice	22.0	14.4	Legumes	27.0	17.8
Canned drinks	21.1	7.8			

Older adults			
Eng & Moy (2011)[21]	NR	NR	Type of fat consumed daily, g/day (% of energy)<br><br>- Saturated fat = 22.7 g/day (10.1% energy)<br>- MUFA = 19.1 g/day (8.52% energy)<br>- PUFA = 12 g/day (5.34% energy)
Asma et al. (2010)[23]	% of recommended no. of servings of carbohydrate-source food groups<br><br>Husbands / wives<br>Grains: 110.4% / 97.3%<br>Vegetables: 69.7 / 68.3<br>Fruits: 59.6 / 49.7	NR	% energy from saturated fat<br><br>- Husbands = 4.3%<br>- Wives = 4.9%
Pon et al. (2006)[15]	CHO foods consumed daily: fruits (71% of subjects); vegetables (90% of subjects)<br><br>Carbohydrate-containing dishes consumed in at least 1 meal during the day (% of subjects)<br>- Rice (in mixed rice, chicken rice, nasi lemak) (100%)<br>- Bread, cookies, cereals (55%)<br>- Noodles soup/similar (30%)<br>- Fried noodles/similar (25%)<br>- Fruits, vegetables (12%)	Protein foods consumed daily<br>(% of subjects)<br><br>- Fish & seafood (36.0%)<br>- Milk (34.7%)<br>- Legumes & products (15.3%)<br>- Poultry (13.1%)<br>- Cheese & yogurt (5.3%)<br>- Meat (4.7%)	NR

- Roti canai, thosai, sandwiches, sweet potatoes (11%)
- Cakes/local pastries (4%)

NR – not reported

* Calculated from Food Balance Sheet data
